# Rapid Metabolome and Bioactivity Profiling of Fungi Associated with the Leaf and Rhizosphere of the Baltic Seagrass *Zostera marina*

**DOI:** 10.3390/md17070419

**Published:** 2019-07-19

**Authors:** Lars-Erik Petersen, Michael Marner, Antje Labes, Deniz Tasdemir

**Affiliations:** 1 GEOMAR Centre for Marine Biotechnology (GEOMAR-Biotech), Research Unit Marine Natural Products Chemistry, GEOMAR Helmholtz Centre for Ocean Research Kiel, Am Kiel-Kanal 44, 24106 Kiel, Germany; 2 Faculty of Mathematics and Natural Sciences, Kiel University, Christian-Albrechts-Platz 4, 24118 Kiel, Germany

**Keywords:** *Zostera marina*, eelgrass, marine fungi, metabolomics, dereplication, HPLC-DAD-MS, bioactivity

## Abstract

*Zostera marina* (eelgrass) is a marine foundation species with key ecological roles in coastal habitats. Its bacterial microbiota has been well studied, but very little is known about its mycobiome. In this study, we have isolated and identified 13 fungal strains, dominated by *Penicillium* species (10 strains), from the leaf and the root rhizosphere of Baltic *Z. marina*. The organic extracts of the fungi that were cultured by an OSMAC (One-Strain–Many-Compounds) regime using five liquid culture media under both static and shaking conditions were investigated for their chemical and bioactivity profiles. All extracts showed strong anti-quorum sensing activity, and the majority of the *Penicillium* extracts displayed antimicrobial or anti-biofilm activity against Gram-negative environmental marine and human pathogens. HPLC-DAD-MS-based rapid metabolome analyses of the extracts indicated the high influence of culture conditions on the secondary metabolite (SM) profiles. Among 69 compounds detected in all *Penicillium* sp. extracts, 46 were successfully dereplicated. Analysis of SM relatedness in culture conditions by Hierarchical Cluster Analysis (HCA) revealed generally low similarity and showed a strong effect of medium selection on chemical profiles of *Penicillium* sp. This is the first study assessing both the metabolite and bioactivity profile of the fungi associated with Baltic eelgrass *Z. marina*.

## 1. Introduction

The common eelgrass *Zostera marina* is a marine flowering plant, which, similar to all seagrasses, evolved from sea to land plants but returned to the sea about 140 million years ago [1]. Today, well-adapted to sea environment, eelgrass meadows dominate the soft sediment shorelines of the temperate northern hemisphere, including Atlantic and Pacific Oceans. Due to its high tolerance to low and variable salinity levels, *Z. marina* represents the most common seagrass in the brackish Baltic Sea [2]. As a foundation species and a key ecosystem engineer, eelgrass plays important ecological roles in primary production, food web support, production of oxygen, organic matter degradation, and recycling of nutrients in coastal environments. *Zostera* beds also provide nursery habitat for many animals (juvenile fish, invertebrates, and other larger animals) and stabilize sediments, preventing coastlines from erosion [3]. The recently completed genome-sequencing project [4] showed that, as part of its re-adaptation process into the marine environment, *Z. marina* had gained genes enabling its tolerance to saltwater. However, it has also lost genes characteristic for terrestrial angiosperms, leading to reduced chemical machinery (e.g., no volatile terpene synthesis), chemical defense, and communication [5]. Nonetheless, a generally low rate of microfouling and biofilm formation in seagrasses [6,7,8] point out defense mechanisms and their ability to control attachment of (micro)foulers. 

It is widely accepted that the microbiome is involved in the maintenance of general health and overall performance of plants. The microbes colonizing the above- or below-ground plant tissues are crucial in fitness, disease resistance, growth, and survival of the host plant [9]. Similar to other angiosperms, *Z. marina* has intimate relationships with both beneficial and harmful microbes in its surrounding environment. Studies on different parts of *Z. marina* suggest their distinct microbial community [2,10,11]. Particularly, the sediment surrounding the roots of eelgrass has been shown to have a unique and rich microflora [5,11,12]. However, almost all these studies have focused on the bacterial microbiota associated with *Z. marina*. Being part of the whole microbiome, the fungi, which are talented producers of antibiotics and many other secondary metabolites (SMs) with diverse bioactivities, may also contribute to overall fitness and chemical defense of any plant/marine organisms. Only a recent study by Kirichuk and Pivkin [13] reported the diversity of cultivable filamentous fungi associated with different parts of *Z. marina* collected in the Sea of Japan (Russia). A few filamentous fungi obtained from *Z. marina* leaves or rhizomes have been studied for isolation of their chemical constituents [14,15]. To our knowledge, no systematic study has been performed on the chemical profiles of cultivable mycobiota of *Z. marina*. In this context, marine fungi are considered not a systematical and taxonomical group, but rather a physiological and ecological, including true marine, as well as facultative strains [16]. 

In this study, we aimed to identify the cultivable mycoflora of the leaf and sediment surrounding the eelgrass roots and investigated their chemical and bioactivity profiles. We used an OSMAC (One-Strain–Many-Compounds)-based culture approach, which led to the isolation and identification of 13 fungal strains from the phyllosphere and the rhizosphere of *Zostera marina* collected from Kiel Fjord (Baltic Sea, Germany). The organic extracts of all fungi that were grown in five different liquid media under both static and shaking conditions were rapidly investigated for their secondary metabolome (using LC-DAD-MS) and screened for their ecologically as well as medically relevant bioactivities (antibacterial, anti-quorum sensing, and anti-biofilm formation).

## 2. Results

### 2.1. Identification and Extraction of Marine Fungi

A total of 13 fungal strains, which were identified based on morphology and ITS sequencing, were isolated. Only one fungus, identified as *Phoma macrostoma*, was obtained from the leaves of *Z. marina*, while the sediment surrounding eelgrass roots afforded 12 fungal strains, 10 of which were successfully identified at species level with 99 and 100% sequence similarities to known species (Table 1). This includes one representative of the genus *Cladosporium* (*Cladosporium langeronii*)*,* one strain of the genus *Trichoderma* (*Trichoderma harzianum*), and 10 *Penicillium* spp., which were assigned to three different species; as *Penicillium antarcticum* (four isolates), *Penicillium atramentosum* (one isolate), *Penicillium atrovenetum* (five isolates). Although different media were used for the isolation campaign, there was no medium selectively increasing the number of obtained strains.

### 2.2. Extraction, Metabolite, and Bioactivity Profile of Fungi Associated with Zostera marina

Culture conditions significantly influence SM profiles of microorganisms. To gain a wide chemical diversity, the isolated fungi were grown in five liquid media differing in their carbon and nitrogen sources and salinity (PC (Potato-Carrot), CA (Casamino Acids), WH (Wickerham15), GPY (Glucose-Peptone-Yeast), and AS (Artificial Seawater)) under both static and shaking (“sh”) conditions. The ethyl acetate (EtOAc) extracts of these cultures were submitted to LC-DAD-MS analyses for rapid chemical profiling. All extracts were tested (at 100 µg/mL concentration) for antibacterial activity against two marine Gram-negative environmental pathogens (the fish pathogen, *Vibrio anguillarum,* and the algal pathogen, *Pseudomonas elyakovii*), the human pathogen *Pseudomonas aeruginosa,* as well as *Aliivibrio fischeri*, which frequently occurs in marine environments [17,18]. The extracts were also evaluated for their biofilm inhibition and anti-quorum sensing efficacy against *Ps. aeruginosa* and *A. fischeri*, respectively. Comparison of antibiotic and anti-QS (quorum sensing)/anti-biofilm activity allows discrimination between these activities for later development of these compounds for different applications, such as antibiotic drugs and biofilm preventing agents, e.g., on medical devices or other sensitive surfaces. 

**Figure 1:**
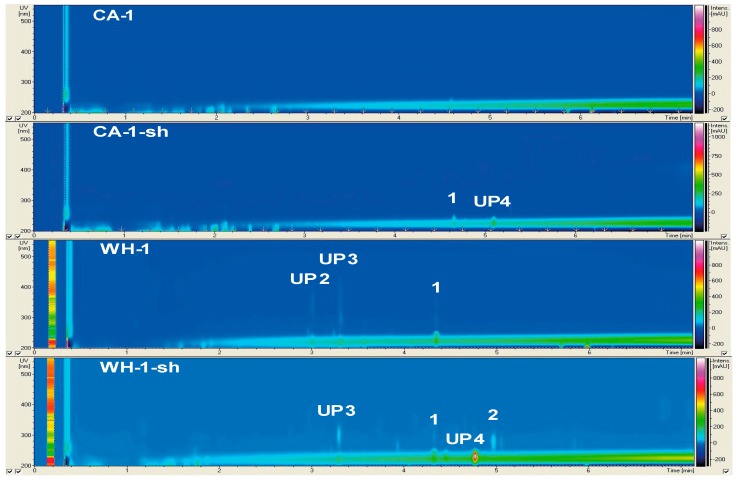
Metabolome profiles of strain 1 (*Ph. macrostoma*) cultured under static and shaking (sh) conditions in selected media. Compounds are displayed as a function of UV absorption (nm) and intensity (mAU) vs. retention time (min). CA: casamino acids medium, WH: Wickerham’s medium. The abbreviations refer to medium (e.g., CA), strain number (1), if used, sh means shaking was used in the culture regime.

**Figure 2:**
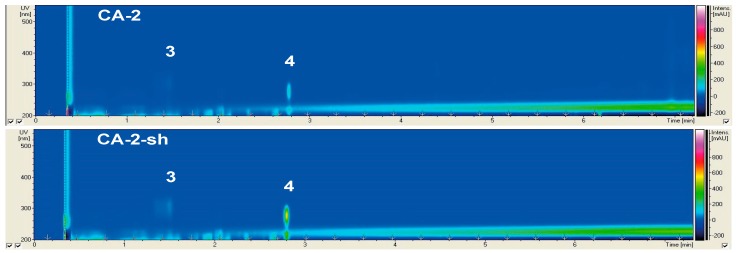
Metabolite profile of strain 2 (*C. langeronii*) cultured under static and shaking (sh) conditions in CA (casamino acids) medium.

**Figure 3:**
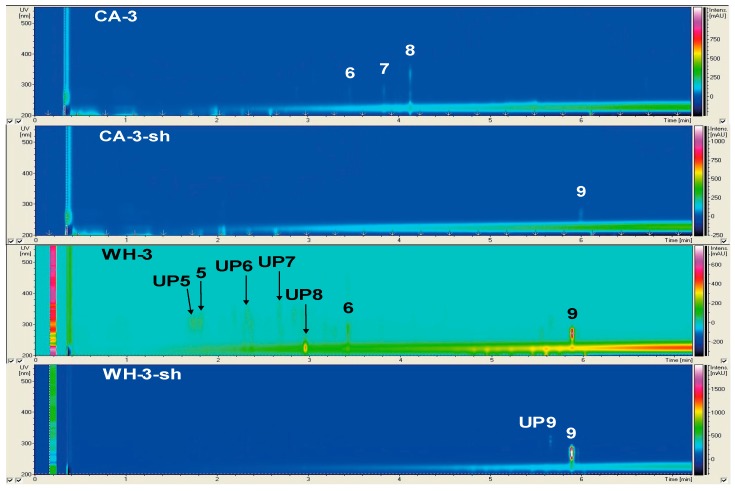
Metabolite profile of strain 3 (*T. harzianum*) cultured under static and shaking (sh) conditions in CA (casamino acids) and WH (Wickerham’s) medium.

**Figure 4:**
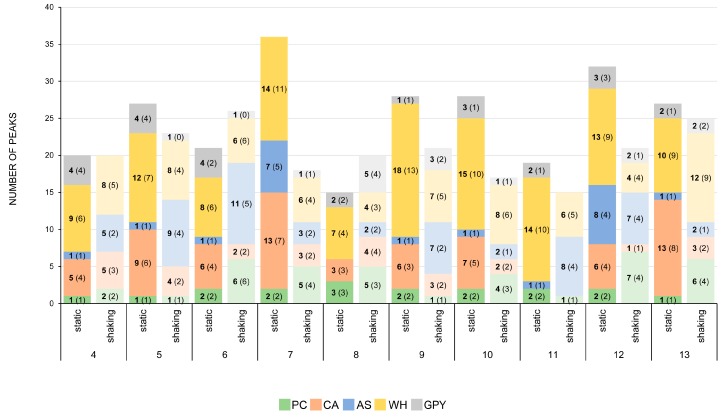
Total peak numbers (TPN) of observed and annotated (in brackets) peaks detected in static and shaking cultures of the ten *Penicillium* strains (e.g., 4 (4) means four peaks were detected, and all four of them were assigned to known compounds). TPNs are sorted by a color code based on the five different liquid media (PC, CA, AS, WH, and GPY). PC: potato-carrot; CA: casamino acids; AS: artificial seawater; WH: Wickerham’s; GPY: glucose-peptone-yeast.

**Figure 5:**
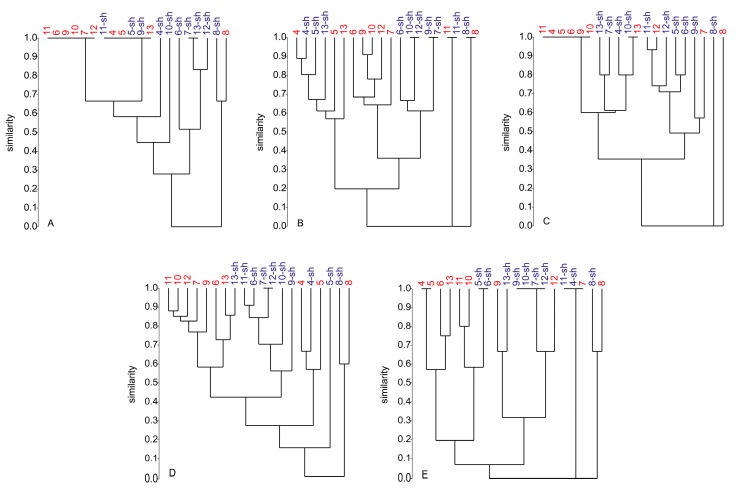
Dendrograms of SMPs (secondary metabolite profiles) of all *Penicillium* strains cultivated under static (blue strain number) and shaking (“sh”, red strain number) conditions in (**A**): PC (potato-carrot), (**B**): CA (casamino acids), (**C**): AS (artificial seawater), (**D**): WH (Wickerham’s), and (**E**): GPY (glucose-peptone-yeast) medium. Strains 4–7: *P. antarcticum*, strain 8: *P. atramentosum*, strains 9–13: *P. atrovenetum* (see Table 1). SMPs of all strains are displayed as a function of similarity based on the Bray-Curtis dissimilarity index.

**Figure 6:**
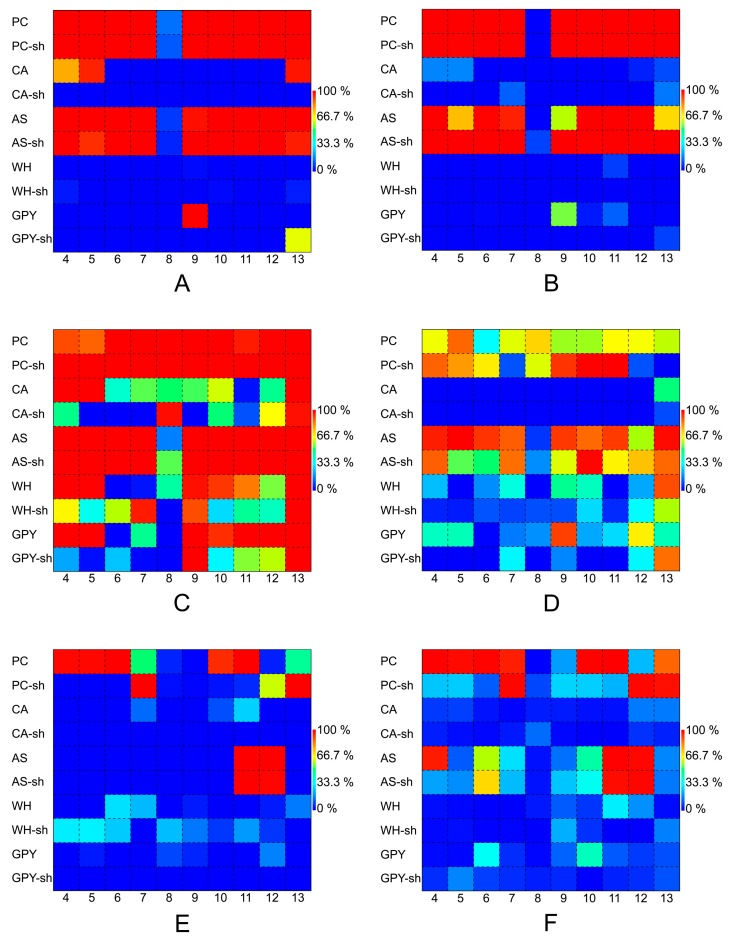
Matrix plots of bioactivities of *Penicillium* spp. (strains 4–13, *x*-axis) grown in different media (*y*-axis) against *V. anguillarum* (**A**), *Ps. elyakovii* (**B**), quorum sensing (**C**), *A. fisheri* (**D**), biofilm formation (**E**), and growth of *Ps. aeruginosa* (**F**). The inhibition levels (%) are displayed in different color intensities (blue: low; red: high). Positive controls: 10 µM chloramphenicol (for **A**, **B**, and **D**); 10 µM furanone (for **C**), and 10 µM polymyxin B (for **E** and **F**). PC: potato-carrot; CA: casamino acids; AS: artificial seawater; WH: Wickerham’s; GPY: glucose-peptone-yeast.

The results of the chemical and bioactivity profiling of non-*Penicillium* strains are presented individually (Figure 1, Figure 2 and Figure 3, Table 2 and Table 3), while the chemical profiles of 10 *Penicillii* are outlined together to allow comparative analyses (Figure 4, Figure 5 and Figure 6). Only a few selected and striking media profiles are displayed as Figures in the main text, while the full chemical profiles of all culture conditions can be found in the Supplementary file. The abbreviations, e.g., CA-1-sh used in Figures (sometimes also in the text) refer to the medium used for culturing (e.g., CA), strain number of the fungi (1), and the application of shaking (sh) in the culture regime.

#### 2.2.1. Secondary Metabolite Profile of *Phoma macrostoma* (Strain 1)

*Phoma macrostoma* was the only fungus isolated from the eelgrass phyllosphere and was the single representative of the genus *Phoma*. Chemical profile of this strain showed significant variations within different media and culture conditions (Figure 1). Table 2 shows the list of compounds detected in all media and culture regimes (for all non-*Penicillium* species, strains 1–3), containing information on their UV absorption, molecular weights, and putative identity. The extracts of both Potato-Carrot (PC) and Wickerham15 (WH) static cultures (shown as PC-1 and WH-1, 1 refers to strain number) contained the unknown peaks **UP2** (UV_max_ 220, 275, 325, 360 nm; *m*/*z* 273.1 [M + H]^+^), **UP3** (UV_max_ 218, 296, 364 nm; *m*/*z* 289.1 [M + H]^+^), and compound **1** (UV_max_ 220, 245, 292, 342 nm; *m*/*z* 229.1 [M + H]^+^). Compound **1** was dereplicated as the furanocoumarin (psoralen) derivative trioxsalen (Table 2). Little to no fungal growth was observed in the statically grown Casamino Acids (CA), Artificial Seawater (AS), and Glucose-Peptone-Yeast (GPY) cultures; hence, their extracts had very poor chemistry (Figure 1, Supplementary Figure S1). In contrast, several compounds were detected in cultures grown under shaking (sh) conditions. The PC-1 medium extract contained three peaks (**UP2**–**3** and **1**), while shaking increased the number of detectable compounds in PC-1-sh to four (**UP1**–**3** and **1**), stimulating the production of another unknown compound **UP1** (UV_max_ 221, 292 nm, *m*/*z* 325.2 [M + H]^+^). In CA-1-sh medium, shaking induced the biosynthesis of **UP4** (UV_max_ 232 nm; *m*/*z* 279.3 [M + H]^+^) and **1**. The strongest influence of shaking was observed in the WH medium. The WH-1-sh extract gained two compounds **UP4** and **2,** which was putatively dereplicated as antibiotic NK-A 17E-233II (UV_max_ 222, 279 nm; *m*/*z* 277.2 [M + H]^+^) but lacked **UP2** (Figure 1). In general, shaking increased the number of detected compounds in strain 1, and PC-1-sh and WH-1-sh cultures exhibited the most diverse chemical composition.

None of the *Ph. macrostoma* extracts had inhibitory activity on environmental pathogens, *V. anguillarum* and *Ps. elyakovii*. Extracts obtained from PC, PC-sh, CA, AS, AS-sh, WH, and WH-sh cultures showed quorum sensing inhibitory (QSI, from 53 to 100% at 100 μg/mL concentration) activity against *A. fischeri*. The extracts of PC and WH slightly (>20%) inhibited the growth of *Ps. aeruginosa*, but not the biofilm formation by this Gram-negative bacterium (Table 3).

#### 2.2.2. Secondary Metabolite Profile of *Cladosporium langeronii* (Strain 2)

*Cladosporium langeronii* was isolated from the rhizosphere of *Z. marina*. As shown in Figure 2, only slight differences were visible in its chemical profile upon the use of different media and culture regimes. Two known compounds (**3** and **4**) were detected in the CA culture extracts of *C. langeronii*, under both static and shaking conditions (Figure 2). The other media were not favored; hence, no compound was detected in the other four media extracts (Supplementary Figure S2). Compound **3** (UV_max_ 221, 289, 310 nm; *m*/*z* 211.1 [M + H]^+^) was annotated as the pyranacetal agistatin D, while compound **4** (UV_max_ 205, 216, 222, 277 nm; *m*/*z* 295.1 [M + H]^+^) was annotated as the orsellinic acid derivative orsellide D (Table 2). Compared to the static CA extracts, compounds **3** and **4** exhibited higher intensities of UV absorption in the CA-2-sh extracts. In general, shaking led minor changes in chemical profiles, and the CA medium appeared to be the best medium for this fungus.

The crude extracts of *C. langeronii* had no inhibitory activity on the growth of *V. anguillarum*, *Ps. elyakovii*, *Ps. aeruginosa,* or the biofilm formation by the latter. Notably, PC, PC-sh, CA, AS-sh, WH, and WH-sh culture extracts strongly inhibited the QS of *A. fischeri* (77%–100% at 100 μg/mL concentration), while the growth of this microorganism was not inhibited (Table 3). 

#### 2.2.3. Secondary Metabolite Profile of *Trichoderma harzianum* (Strain 3)

*Trichoderma harzianum* was the second strain isolated from the rhizosphere of *Z. marina*. The metabolite profile of this strain possessed distinct diversity within different media and culture regimes (Figure 3, Supplementary Figure S3, Table 2). The static cultures of strain 3 produced altogether nine compounds (**UP5**–**8** and **5**–**9**). With seven detected metabolites, statically grown WH culture yielded the chemically richest extracts. It contained four unknown compounds, **UP5** (UV_max_ 215, 303 nm, *m*/*z* 327.3 [M + H]^+^), **UP6** (UV_max_ 218, 297 nm, *m*/*z* 312.2 [M + H]^+^), **UP7** (UV_max_ 220, 266, 276, 287 nm; *m*/*z* 393.1 [M + H]^+^), and **UP8** (UV_max_ 226 nm; *m*/*z* 245.2 [M + H]^+^). The compounds **5** (UV_max_ 215, 306 nm; *m*/*z* 311.3 [M + H]^+^), **6** (UV_max_ 223, 275, 288 nm; *m*/*z* 599.3 [M + H]^+^), and **9** (UV_max_ 223, 261, 271, 281; *m*/*z* 282.2 [M + H]^+^) were putatively identified as orsellide C, the cyclotetrapeptide cyl-2, and 2′-deoxyribofuranosylguanine (2N-Me), respectively. Three peaks were detected in static CA-3 cultures (**6**–**8**, Table 2). Compounds **7** (UV_max_ 223, 280 nm; *m*/*z* 316.1 [M + H]^+^) and **8** (UV_max_ 227, 269, 334 nm; *m*/*z* 282.1 [M + H]^+^) were dereplicated as quinolinone B and harzianopyridone alkaloids, respectively. The only detectable compound in the extracts of the static PC-3 and the shaken CA-3-sh cultures was compound **9**. No peaks were detected in the statically grown AS and GPY cultures. In general, shaking suppressed the number of compounds produced by strain 3. Strikingly, the overall number of detected peaks in WH cultures declined from nine to two upon shaking (**UP9** and **9**), while, an unidentified compound **UP9** (UV_max_ 222, 280, 292, 305, 319 nm; *m*/*z* 626.2 [M + H]^+^) and **9** were visible in WH-3-sh extract. Overall, static culture regime using WH medium provided the most favored conditions for yielding the richest secondary metabolite profile (SMP) of *T. harzianum*.

Several *Trichoderma harzianum* crude extracts, namely PC, CA, CA-sh, AS-sh, WH, and WH-sh, showed QSI activity against *A. fischeri* (70% to 97% inhibitory capacity). Also, the growth of *A. fischeri* was inhibited by extracts derived from the PC (70%) and WH (46%) media cultures of *T. harzianum* (Table 3).

#### 2.2.4. Secondary Metabolite Profile of *Penicillium* spp. (Strains 4–13)

Due to their abundance, the chemical profiles of all 10 *Penicillium* strains associated with the rhizosphere of *Z. marina* were explored together. Their SM profiles (cultivated in five different liquid media under both shaking and static conditions) were represented in a comparative manner rather than individually. Supplementary Table S1 contains detailed information on UV absorptions, molecular weights, and putative identities of compounds detected in *Penicillium* extracts in all media and culture regimes. Among 69 compounds detected in *Penicillium* extracts in total, 46 were assigned to known compounds (Supplementary Figure S14 and Supplementary Table S1). Figure 4 illustrates the total peak number (TPN), as well as the number of putatively dereplicated compounds, for each *Penicillium* strain fermented under both static and shaking conditions. These numbers represent the sum of detected compounds versus the sum of annotated compounds in all five media. 

Figure 4 shows that the most productive *Penicillium* strain under static conditions (all peaks detected in all static cultures counted together) was strain 7 (*P. antarcticum*) with 36 detected peaks in all media, followed by strain 12 (32 peaks), 9 (28 peaks), 10 (28 peaks), 5 (27 peaks), 13 (27 peaks), 6 (21 peaks), 4 (20 peaks), 11 (19 peaks), and 8 (15 peaks). In most cases, approximately one-quarter of all detected peaks in extracts of static cultures remained unknown, while three-quarters were dereplicated and assigned to known compounds (see Supplementary Table S1). In general, extracts deriving from statically grown WH and CA cultures were found to be richer in SM than those grown statically in PC, AS, and GPY cultures (Figure 4). The highest TPN in static WH medium cultures was observed in the extract of strain 9, exhibiting 18 detected peaks. Also, strains 7, 10, 11, and 12 produced many compounds in static WH medium. In statically incubated CA cultures, the highest TPNs were detected in strains 7 and 13, both containing 13 peaks. In summary, under static conditions, WH and CA media provide the richest metabolite profiles for *Penicillium* strains (Figure 4). In contrast, PC, AS, and GPY media yield poor SM profiles in static cultures. Notably, strains 7 and 12 produced high numbers of compounds in all media, including the nutrient-poor AS medium, under static conditions.

Under shaking conditions, the most productive *Penicillium* sp. was strain 6 (*P. antarcticum*), which contained altogether 26 peaks detected in all media extracts. This strain was followed by strain 13 (25 peaks), 5 (23 peaks), 9 (21 peaks), 12 (21 peaks), 4 (20 peaks), 8 (20 peaks), 7 (18 peaks), 10 (17 peaks), and 11 (15 peaks). Most compounds found in the extracts of shaking cultures were assigned to known compounds, especially those in the extracts of CA and WH cultures (e.g., strains 6, 7, 8, 10, and 13). However, many compounds remained unknown in the shaking culture extracts of AS medium (e.g., strains 5, 6, 9, 11, and 12). In comparison to static conditions, shaking induced the chemical diversity of the AS and PC cultures, while it was mostly vice versa in the CA and WH cultures. The best performer in shaking AS cultures was strain 6 with a TPN of 11, five of which were annotated to known compounds (Supplementary Table S1). Strains 5, 9, 11, and 12 possessed rich SMPs with many unknown compounds in AS-sh cultures. The highest TPN in shaking PC cultures was found in the extracts of strain 12, possessing seven peaks. In contrast to AS-sh cultures, the peaks in PC-sh cultures were assigned to known compounds (Supplementary Table S1). Except for strains 7, 8, 9, and 13, shaking led to an unfavorable effect in chemical diversity of GPY culture extracts. These findings show that the effect of aeration (shaking) on the SMP of most *Penicillium* strains is medium-dependent. However, most strains exhibited overall similar sums of TPNs, except for strains 7, 8, and 12. 

Although identified as same species, strains 4–7 (*P. antarcticum*), strain 8 (*P. atramentosum*), and strains 9–13 (*P. atrovenetum*, see Table 1) did strongly vary in their SMPs, and their response to stimuli through culture conditions was strain-dependent (Supplementary Figures S4–S13). Hierarchical cluster analysis (HCA) based on Bray-Curtis similarity was applied to compare the similarity of the chemical profiles of all *Penicillium* strains (based on presence/absence of compounds in different culture extracts) grown in five different media under static and shaking regimes. In this analysis, a similarity value of 1.0 indicates that two (or more) strains have 100% identical SMPs (producing a horizontal line at the top), while a similarity value of 0 shows that their SMPs are completely unrelated (0% similarity, producing a horizontal line at the bottom). Similarly, a value between 0 and one implies partial relatedness among SMPs of the respective strains. Each branch in a dendrogram represents one SMP of a specific strain (or more, if similar) cultivated under two culture regimes. Therefore, strongly branched dendrograms indicate a high variety of SMPs of respective strains in one media and low chemical relatedness between the *Penicillium* strains. In contrast, weakly branched dendrograms indicate *vice versa*. Hence, HCA allowed quick assessment of the impact of culture media and culture regimes in a color-coded manner (statically grown cultures in blue, shaken in red) on the chemical diversity of all *Penicillium* strains. The resulting dendrograms are illustrated in Figure 5. In general, cultivation in CA and WH media (Figure 5B,D) resulted in more complex dendrograms than those in PC, AS, and GPY media. Clusters of the CA and WH cultures were considerably branched, and only small groups of identical SMPs were formed (Figure 5B,D). In contrast, clusters of PC, AS, and GPY cultures exhibited fewer branches but contained more and larger groups of identical similarity. This indicates that SMPs of PC, AS, and GPY cultures are not as diverse as the CA or WH cultures and more sensitive to the culture regime employed. Notably, the strain 8 (*P. atramentosum)* appeared to be chemically distant to all other *Penicillium* strains in all five media and both culture regimes, while the remaining *Penicillium* strains shared similarities of at least 0.05 (Figure 5E). 

Specifically, in the PC medium (Figure 5A), the similarity between the static (8) and shaken (8-sh) cultures of strain 8 was approx. 0.65, i.e., showed 65% similarity. However, SMP similarity of these cultures with other *Penicillium* strains was 0. As shown in Figure 5A, strains 11, 6, 9, 10, 7, and 12 formed a large cluster with a similarity value of 1.0 in static PC cultures. A second cluster with 100% similarity was obvious between strains cultured under both static and shaking conditions (11-sh, 4, 5, 5-sh, 9-sh, 13). Both clusters had a similarity value of approx. 0.65. A third small cluster in Figure 5A with identical SMP was formed by 7-sh and 13-sh cultures. All other strains showed similarities between 0.25 and 0.85, indicating major differences but also overlaps in the SMPs. 

The dendrogram of all static and shaking *Penicillium* CA culture extracts (Figure 5B) was highly branched, indicative of high chemical diversity. As shown in Figure 5B, four small groups with a 100% similarity were formed, each consisting of two representatives (10-sh/12-sh; 9-sh/7-sh; 11/11-sh; 8/8-sh). For strain 8, the static and shaking conditions resulted in an identical SMP, indicating that the culture regime had no impact on its SMP in CA medium. Again, the SMP of strain 8 was chemically unrelated (similarity of 0) to all other *Penicillii*. The same applies to the SMP of strain 11 in CA medium. The SMPs of all other strains were highly clustered and showed similarities between 0.2 and 0.9. Therefore, some compounds were common in all *Penicillium* isolates in CA medium, and others were exclusive to specific strains. 

In the AS medium (Figure 5C), culture regime had a strong impact on the SMP of strain 8, with 0% similarity. Both 8 and 8-sh cultures were yet unrelated to the remaining *Penicillium* strains. Statically grown strains 11, 4, 5, 6, 9, and 10 formed a big cluster with 100% similarity. The same applies to strains 10-sh and 13. The other strains exhibited similarity values ranging from 0.35 to 0.95 (Figure 5C), indicating increased variability in their SMPs upon shaking.

The dendrogram of WH cultures (Figure 5D) was highly branched. It contained many clusters and exhibited only one small cluster, consisting of strains 7-sh and 12-sh, with 100% identical SMP. The strain 8 was again chemically unrelated to all other *Penicillium* spp. but shared approx. 60% SMP similarity between static and shaking cultures. Most of the strains showed similarity values between 0.15 and 0.9 in different culture regimes, indicating high chemical diversity. 

In the GPY medium (Figure 5E), the static and shaking culture regimes led to a 65% SMP similarity in strain 8, that was again 100% distant from the remaining *Penicillium* strains. A few clusters with 100% similarity were visible, i.e., 4/5; 5-sh/6-sh, 9-sh/10-sh/7-sh/12-shs and 11-sh/4-sh/7s in the dendrogram (Figure 5E). The latter cluster, as well as the 8/8-sh cultures, had no chemical relatedness to other *Penicillii*. The remaining strains formed two big sub-clusters with a similarity value of 0.05. These results highlight the impact of culture regime on the chemical diversity of *Penicillium* strains in GPY medium. 

#### 2.2.5. Bioactivity of *Penicillium* Species

The crude extracts of all *Penicillium* isolates were screened against the same panel of microorganisms for their antibacterial activity, as well as their inhibitory effects on quorum sensing (QSI) and biofilm formation. To allow clear and quick assessment of the bioactivity profile of different *Penicillium* spp., their bioactivities were displayed using matrix plots (Figure 6). Inhibition rates (%, in comparison to standard antibiotics) of fungal extracts are displayed as a function of color intensity ranging from blue (low inhibition) to red (high inhibition). The matrix plots of bioactivities revealed similar patterns of bioactivity against *V. anguillarum* and *Ps. elyakovii* for all *Penicillii* (Figure 6A,B). Almost all PC, PC-sh, AS, and AS-sh media extracts, except for those of strain 8, potently inhibited *V. anguillarum* and *Ps. elyakovii*. The crude extracts of WH, CA, and GPY cultures (static and shaking) were inactive against both pathogens. The only exceptions were the strain 9 (in GPY medium) and strains 4, 5, and 13 (in CA medium, Figure 6A,B), all incubated in static conditions. Figure 6C,D displays the activity of all *Penicillii* extracts for QSI and antibacterial activity against *A. fischeri*, respectively, as matrix plots. The majority of the crude extracts displayed QSI activity, while only some inhibited the growth of *A. fischeri*. Similar to the activity observed against *V. anguillarum* and *Ps. elyakovii*, almost all PC/PC-sh and AS/AS-sh culture extracts exhibited potent anti-quorum sensing activity. The static cultures of a few additional strains cultured in CA (e.g., strains 4, 5, and 13), CA-sh (strains 8, 13), WH medium (strains 4, 5, 9, 10, and 13), GPY (strains 4, 5, 9–13), and GPY-sh (strains 9 and 13) also showed high QSI potential (Figure 6C). Several extracts obtained from the static AS media (4, 5, 6, 9, 11, and 13) and few extracts obtained under shaking conditions, i.e., strains 9, 11 (PC-sh), and 10 (PC-sh and AS-sh), inhibited the growth of *A. fischeri*. None of the CA and CA-sh extracts showed reasonable inhibition of *A. fischeri*. 

The bioactivity of *Penicillium* strains against the growth and biofilm formation by *Ps. aeruginosa* is depicted in Figure 6E,F, respectively. The highest anti-biofilm activity against *Ps. aeruginosa* was exerted by four strains grown in static conditions in PC medium (4, 5, 6, 10, and 11) and two strains (7, 13) grown in PC-sh (Figure 6E). The strains 11 and 12 grown in both AS and AS-sh media had high anti-biofilm potential (Figure 6E). Almost all these extracts, as well as strains 4 (in AS), 10 and 11 (in PC), and 12 and 13 (in PC-sh), displayed antibacterial activity against this Gram-negative bacterium (Figure 6F). In comparison to former strains 1–3, *Penicillium* spp. overall appeared to have higher bioactivity potential, not only showing QSI and anti-biofilm formation but also antibacterial activity against Gram-negative bacteria.

## 3. Discussions

### 3.1. Fungi Associated with the Leaf and Rhizosphere of Baltic Z. marina

In continuation of our research interest into *Z. marina* from the Baltic Sea [6,8], we have investigated fungal communities associated with its phyllosphere and rhizosphere. Dissected eelgrass specimens and seagrass rhizosphere sediment samples led to the isolation of 13 fungi, belonging to four different genera and six different species. Interestingly, only one isolate (*Phoma macrostoma*) derived from the *Zostera* leaves. This is lower than that reported in previous studies. Newell (1981), who incubated the green and brown (dead) leaves of *Z. marina* from Chesapeake Bay (USA) in seawater without an additional carbon source identified fungi belonging to six different genera (by microscopic observation) as well as living mycelia without identifiable propagules [27]. The only detailed mycobiome study performed on *Z. marina* is from Rifovaya Bay (Sea of Japan) by Kirichuk and Pivkin (2015) that reported 16 fungi from the phylloplane (specifically the leaves, without stems, flowers, or fruits). They belong to diverse genera, i.e., *Acremonium, Arthrinium, Aspergillus, Beauveria, Cladosporium, Trichoderma*, and *Penicillium*, the latter being the most abundant one (seven isolates). To our knowledge, no previous study reported the isolation of a *Phoma* strain from *Z. marina* leaves. The discrepancy between our work and that of Kirichuk and Pivkin (2015) may stem from potential temporal variations (the different time/season for sampling/isolation of the fungi that was unfortunately not stated), spatial differences (different collection site, i.e., Rifovaya Bay, Sea of Japan versus Baltic Sea), as well as their larger sample size (four stations) [13]. In the present study, the seagrass rhizosphere afforded 12 filamentous fungi belonging to genera *Cladosporium*, *Trichoderma,* and *Penicillium*. With 10 isolates, the genus *Penicillium* dominated the sediment mycobiome. The previous work by Kirichuk and Pivkin (2015) reported similar numbers (16) from the eelgrass rhizosphere, also predominantly composed of *Penicillium* spp. [13]. Notably, the same authors have inoculated their samples on Agar wort (prepared with seawater containing unhopped beer wort) or directly on sterile filter papers [13]. Herein, we used five different agar media in Petri dishes, which is regarded as the most suitable inoculation method for fungal strain isolation [28]. The other experimental details (e.g., potential dilution, temperature, total inoculation/incubation time, identification other than morphological traits) are absent in their report to enable comparisons. The common finding in these two studies is the dominance of *Penicillium* species in rhizosphere samples. 

It has been shown that the microbiome (bacterial communities) of the surface (epiphytic), inner tissues (endophytic), and sediments surrounding the roots (rhizopheric) of seagrasses are quite distinct. In particular, the sediment microbiome has been reported to represent a unique and dynamic microsystem [5,11,12,29]. Seagrasses grow in anoxic and sulfidic sediments as a result of deposition of large amounts of organic material in seagrass meadows. The plant-driven gradients of oxygen and dissolved organic carbon determine the distinct microbiome of the rhizosphere [30,31,32]. The availability of various electron acceptors (sulfate) and the exudates released by the seagrass roots promote bacterial growth and microbial processes, hence, allowing a unique niche of microecosystem with complex microbial assemblages around the rhizosphere. Although such high carbon stocks may represent a suitable environment for fungal growth, very little is known on fungal assemblages in *Zostera* rhizosphere (or in the other parts of *Zostera* spp.) or how they modulate the plant microbe-interactions, thereby possibly affecting the health of the plant. Furthermore, no attempt has been done for chemical and bioactivity profiling on endophytic and rhizospheric fungal communities of *Z. marina*. This has been the main aim of this study. 

### 3.2. Dereplication of Secondary Metabolite Profiles of Z. marina Associated Fungi

Despite the advancement of modern analytical instruments and dereplication methods, the identification of known compounds in complex natural extracts remains a challenging endeavor. To decipher the chemical potential of *Z. marina* associated fungi, we herein followed an OSMAC approach using different media and culture regimes to stimulate the production of putatively novel SMs [33]. For a rapid assessment of the chemical diversity produced in *Z. marina* associated fungi, we compared information gained from HPLC-DAD-MS measurements (UV/VIS absorption and molecular mass) with entries in large public and commercial databases to conduct dereplication of known compounds. 

Strain 1 was genetically identified as *Phoma macrostoma*. The genus *Phoma* has never been isolated from *Z. marina* before, although *Phoma* spp. are common in Nature, including terrestrial and marine hosts and marine (Arctic) sediments [34,35,36]. Chemically, the crude extracts of this strain displayed moderate chemical diversity. Analyses of the SMP of this strain showed PC and WH media to be the most suitable. The fungal growth, in the static CA, AS, and GPY cultures, was poor, so was the chemical profile. SMPs of the crude PC and PC-sh culture extracts looked alike except for the presence of **UP1** with a weak chromophore in PC-sh. **UP2** and **UP3** occurred in static PC and WH cultures, and the same applied for **UP4** in shaken CA and WH cultures. Two known compounds were identified as trioxsalen (**1**) and “NK-A 17E-233II” (**2**). Trioxsalen (aka 4,5′,8-trimethylpsoralen) is a phototoxic furanocoumarin originally isolated from domestic celery infected with the phytopathogenic fungus *Sclerotinia sclerotium* [19]. It is used for the phototherapeutic treatment of vitiligo [37] and is cytotoxic against human melanoma cells [38]. The second compound “NK-A 17E-233II” is obtained from a marine *Phoma* sp. [20] with reported antitumor activity. In the present study, the extracts of PC, PC-sh, WH, WH-sh, and CA-sh cultures exhibited high QSI activity (Table 3). 

The fungal genus *Cladosporium* has been reported from *Z. marina* by Kirichuk and Pivkin [13] and Newell [27]. In general, *Cladosporium* spp. are widespread and have been isolated from numerous marine sources, including algae [39]. The SMP of strain 2 cultivated in five static and shaken media was overall poor. The crude extracts of CA-2 and CA-2-sh cultures contained only two peaks, which were identified as agistatin D (**3**) and orsellide D (**4**) from their UV_max_ and distinct masses (Table 2). This is the first report indicating the presence of agistatin D and orsellide D in the genus *Cladosporium*. Agistatin D is a pyranacetal originally isolated from a *Fusarium* sp. that inhibits the cholesterol biosynthesis [21]. Orsellide D is the deoxyhexose glycoside of orsellinic acid (2,4-dihydroxy-6-methylbenzoic acid) that was extracted from an alga-associated *Chaetomium* sp. It shows moderate antibacterial activity against *Escherichia coli*, *Bacillus subtilis*, and *Staphylococcus aureus* [22]. The chemical profile analyses showed that variations in culture conditions did not much affect the SM production of this strain, although there was a preference for CA medium. The crude extracts of both CA and CA-sh cultures containing agistatin D and orsellide D did not inhibit any of the test microorganisms but inhibited QS with variable potency (95% and 30% inhibition of luminescence in *A. fischeri*, respectively, see Table 3). It might be of interest to optimize the static culture conditions of this strain to induce the biosynthesis of other compounds and test their QSI potential. 

Members of the *Trichoderma* genus (strain 3) have previously been isolated from *Z. marina* specimens [13], as well as from the Mediterranean seagrass *Posidonia oceanica* [40,41]. *Trichoderma* spp. are common and reported from various marine sources, including algae [42] and terrestrial plant rhizosphere [43]. Strain 3 showed strong medium preferences for static and shaken CA and WH media, while no compounds were detected in AS and GPY media extracts. From 10 peaks detected in different cultures of strain 3, only five were putatively dereplicated. Peak **5** was identified as orsellide C with reported antibacterial activity [40]. This compound was only detectable in the static WH culture. **UP5** and **UP6** might be potential new derivatives of orsellide C due to shared UV absorption. The compound **6,** common to both static WH and CA cultures, was annotated as cyclotetrapeptide (Cyl-2) that originates from the fungal phytopathogen *Cylindrocladium scoparium* [23] and inhibits the growth of lettuce and rice seedlings [44]. Peak **7** was identified as quinolone B, an alkaloid originally isolated from a soil-derived *Penicillium* sp. with insecticidal and nematocidal activity [24,45]. Peak **8** was assigned to harzianopyridone (**8**), a small pyridine derivative with antimicrobial activity [46], including phytopathogens [25]. Compounds **7** and **8** were solely detected in static CA cultures. Peak **9** was identified as 2’-deoxyribofuranosylguanine (2N-*Me*), a primary metabolite expressed in PC, CA-sh, WH, and WH-sh cultures. All unknown compounds were solely detected in static WH cultures, while **UP9** was observed only in the WH-sh extract. The extracts of strain 3 lacked antibacterial activity, except for the static PC and the WH extracts that inhibited *A. fischeri*. Almost all extracts, particularly those deriving from CA, CA-sh, WH, and WH-sh cultures, strongly inhibited QS (Table 3). 

With ten isolated strains, the fungal genus *Penicillium* showed the highest abundance for *Z. marina* (Table 1). The strains (4–13) belonged to three species, *P. atramentosum* (strain 8), *P. antarcticum* (strains 4–7), and *P. atrovenetum* (strains 9–13). In general, *Penicillium* spp. are widely distributed fungi found in all moderate environments [47]. They are also the chemically best-studied genus from which various types of SMs, including polyketides, terpenoids, peptides, and alkaloids, have been reported [48]. Still, they continue providing new SMs [49,50], often due to sampling campaigns conducted at extreme environments, e.g., deep-sea [51]. Afiyatullov and colleagues [14] reported the isolation of a new phthalide derivative from *Penicillium claviforme*, a surface associated fungus of *Z. marina* sampled in the Sea of Japan. The same authors [15] isolated four new eudesmane-type sesquiterpenes from *Penicillium thomii*, from *Z. marina* sampled from the same location. Three of the four tested compounds induced a significant down-regulation of nitric oxide production in murine macrophages. To our knowledge, none of the previously mentioned studies conducted a systematic approach to investigate the chemical profiles and bioactivities of cultivable mycobiota of *Z. marina*. In this study, a total number of 69 compounds with different UV_max_ and molecular masses were detected from all 10 *Penicillium* spp. Of these, 46 (approx. 70%) were successfully dereplicated, while 23 peaks (approx. 30%) remained unidentified. Known compounds belonged to chemical classes of alkaloids, polyketides, and terpenoids (Supplementary Table S1). Interestingly, this annotation ratio applies largely to every single strain regardless of aeration and independent of taxonomy. As mentioned above, strain 8 appeared to be very unique in chemistry (Figure 5 and Supplementary Table S1). All twelve compounds, identified from this strain, were species-specific, with the only exception being the terpenoid molecule andrastin A that also occurs in strain 4 (*P. antarcticum*). Several other dereplicated compounds, as well as potential new (unknown) compounds (Supplementary Table S1) also existed in single strain or a single species. For example, atrovenetin (**43**) was specific to *P. atrovenetum* and present in all *P. atrovenetum* strains 9–13, but no other *Penicillium* species contained this compound. However, the majority of the compounds existed in multiple *Penicillium* samples, excluding a general strain or species-specificity. For example, all *Penicillii*, except for strain 8, produced compound **10** in static and shaken PC and AS media. Compound **10** was dereplicated as patulin, a mycotoxin that has been isolated from a variety of *Penicillium* spp. [52] with a broad range of bioactivities (see Section 3.4). Dictyosphaeric acid B (**36**), a polyketide, was common to all *P. antarcticum* and *P. atroventumum* strains (Supplementary Table S1). Another mycotoxin, dereplicated in almost all extracts of the *Penicillium* strains, was compound **54**. It was assigned to aflatoxin M_1_, another mycotoxin that shows cytotoxicity and genotoxicity (e.g., DNA damage and gene mutation) in mammalian cells, insects, lower eukaryotes, and bacteria [53]. Besides mycotoxins, the *Z. marina* associated *Penicilli* appeared to produce compounds with a broad variety of activities. Examples include the lactone cytosporone E (**18**) from a *Cytospora sp.* [54], the terpenoid hongoquercin A (**24**) from an unidentified fungus [55], and the alkaloid bionectin B (**37**) that has been reported from the soil or plant-derived fungus *Bionectra byssicola* [56]. Reportedly, all these compounds show antibacterial activities. Furthermore, podosporin A (**35**), a quinone from the ascomycete *Podospora decipiens* [57], and talaroconvolutin B (**55**), a tetramic acid derivative originating from another fungus *Talaromyces convolutus* [58], are known to exhibit antifungal activity. Compound **38** (tubingensin A or B from *Aspergillus tubingensis*) has been shown to display antiviral activity against herpes simplex virus type 1 [59,60]. Compound **43** was dereplicated as atrovenetin, a polyketide isolated from *Penicillium paraherquei* with antioxidant activity [61]. Adding to this list of bioactivities, the terpenoids territrem C (**33**) from *Aspergillus terreus* [62], penitrem E (**49**) from *P. crustosum* [63], and compound **53** (penitrem A or pennigritrem from *Penicillium* sp.) [64] are mycotoxins with tremorgenic activity in mice [63]. Some compounds that are unique to the chemically distinct strain 8 (*P. atramentosum*) are known for their cytotoxicity and anticancer activity. Examples include globosumone B (**12**), an ester of orsellinic acid isolated from the fungus *Chaetomium globosum* with moderate activity against multiple tumor cell lines [65], or compound **51** (penicillenol A1 or A2), which is an anticancer tetramic acid derivative originating from a *Penicillium* sp. [66]. With their diversity and broad-spectrum bioactivities, *Penicillium* spp. that are abundantly found in *Z. marina* rhizosphere may be beneficial for the protection of the eelgrass roots. 

### 3.3. The Influence of Media and Culture Regime on the Secondary Metabolome of Z. marina Associated Fungi

The OSMAC approach has proven to be a very successful strategy for the discovery of novel SMs. In this study, the selection of culture media significantly influenced the chemical diversity of *Z. marina* associated fungi. Figure 1, Figure 3, Figure 4, and Figure 5D indicate that WH medium provided chemically diverse SMPs in all associated fungi. Dereplication of all fungal extracts annotated metabolites unique to every single medium (independent of culture regime), i.e., WH with 26 unique SMs, AS with 12 unique SMs, CA with 11 unique SMs, PC with 10 unique SMs, and GPY with two unique SMs (Supplementary Table S1). The medium-dependent occurrence of SMs may suggest the activation of silent biosynthetic gene clusters by variation of culture conditions. Figure 1, Figure 3, Figure 4, and Figure 5D indicate that WH medium induced chemically diverse SMPs in all associated fungi. WH medium is rich in nutrients, containing high amounts of carbon sources (glucose and malt) and nitrogen sources (peptone and yeast), as well as sea salt mixture as a salt source. *Penicillium* spp. generally respond well to changes in growth medium composition, especially to salinity and salt sources [67,68]. The influence of the C source on the SM production has also been reported. Rodríguez-Ortiz and coworkers (2010) observed sucrose to stimulate the production of the antibiotic metabolite bikaverin in *Fusarium fujikuroi* [69]. Another study by Fan and colleagues (2019) showed that a specific family of linear aminolipids was exclusively detected in the sucrose-containing medium [70]. Besides carbon, the choice of the nitrogen source also plays an important role. An early study on *Aspergillus flavus* reported that the absence of yeast extract in the respective growth medium led to the silencing of mycotoxin production [71]. However, with 18 produced compounds, strain 9 (*P. atrovenetum*) was the best producer in static WH medium. The dendrogram of WH medium (Figure 5D) further emphasized the suitability of this medium for high SM production in *Penicillium* strains. In contrast to PC, AS, and GPY media (Figure 5A,E), the dendrogram of WH medium was highly branched, indicating a high chemical diversity. The AS medium is poor in nutrients, containing low amounts of glucose as a carbon source without additional N source. Therefore, the fungal growth was generally weak, and the AS medium extracts were chemically poor. Nevertheless, cultivation of the *Penicillium* strains in AS medium resulted in chemically diverse extracts (see Figure 5C). Strain 6 produced 11 SMs with only five known compounds and was the best producer in this medium under shaking conditions. The high number of unknown compounds in shaken AS cultures (see Figure 4) suggests that fungal strains can produce many putatively new natural products in this medium. The CA medium is rich in nitrogen sources (hydrolyzed casein and yeast extract) and supplemented with a sea salt mixture as a salt source and dipotassium phosphate. The PC medium contains a complex mixture of C and N sources without extra salt source. The highest TPN (seven compounds) in PC medium was achieved by strain 12 (*P. atrovenetum*) under shaking conditions. GPY medium contains glucose as a carbon source and peptone and yeast as a nitrogen source, all in low amounts. The highest TPN (5) in GPY medium was achieved by strain 8 under shaking conditions (Figure 4). In contrast to the three media mentioned above, PC and GPY media did not contain the sea salt mixture. These findings imply the necessity of salt for the cultivation of diverse SMs in *Z. marina* associated fungi. 

The differences in chemical production under different culture conditions might also stem from changes in physical parameters. In general, shaking facilitates aeration and the uptake of oxygen [72]. This is necessary to provide adequate mixing and mass transfer, especially when fungal cells grow in a freely dispersed form, which results in a non-Newtonian broth with high viscosity [73]. In the present study, we applied shaking at 120 rpm to liquid cultures. Bigelis and coworkers (2006) researched fungal antibiotics and showed that fungi formed dispersed mycelia under shaking conditions but mycelial mat under static conditions [74]. Chemical production was significantly affected by different mycelial growth conditions. In the current study, we observed shaking led to the production of more (unknown) compounds in the AS medium (Figure 4). The average number of SMs strongly increased for all *Penicillii* cultured under shaking regime in AS medium. This positive effect of shaking was not observed for the other four media. For example, cultivation in shaken CA medium resulted in significantly lower numbers of detected SMs in all *Penicillium* strains compared to static CA medium. The average number of compounds of all *Penicillium* strains cultured under shaking conditions in CA media, therefore, strongly sank. In any case, the results indicate that the availability of higher levels of oxygen might be a critical factor for the production of SMs in different media. Guo and co-workers (2013) have previously reported the expression of specific fungal SM in a *Penicillium* sp., where the shift from static to shaking conditions led to the discovery of five new sorbicillinoids [75]. Noteworthy is that strain 8 generally exhibited completely different SMPs than the other *Penicillii* strains in both static and shaking regimes (Figure 5A–E).

### 3.4. Bioactivities of the Extracts of Z. marina Associated Fungi

All fungal extracts were tested for bioactivity against *V. anguillarum* and *Ps. Elyakovii,* as well as against QS in *A. fischeri* and biofilm formation of *Ps. aeruginosa*. *V. anguillarum* is a Gram-negative bacterium and causative agent of vibriosis, which is pathogenic to a variety of aquatic organisms, such as fish and crustaceans, with importance in aquaculture industry [76,77,78,79]. *Ps. elyakovii* is another Gram-negative bacterium that is isolated from spot-wounded fronds of the brown alga *Laminaria japonica* and is believed to cause this disease [80]. Screening for extracts containing active compounds for purification of chemicals with application potential in aquaculture is, therefore, of interest. Multi-drug resistant pathogenic bacteria are on the rise worldwide, and it is of great urgency to develop novel therapeutic measures. A promising approach is to manipulate the cell-to-cell communication (known as QS), which is crucial for microfouling of biological surfaces that increase the vulnerability of marine organisms to water-borne pathogens. QS inhibition is also being regarded as a starting point for the discovery and development of new antibiotics and antipathogenic drugs [81]. In this study, the marine Gram-negative bacterium *A. fischeri*, emitting bioluminescence through QS [82], was used for anti-quorum sensing assays. Because QS is directly linked to bacterial population-density, tests for growth inhibition in *A. fischeri* were performed as well. This bacterium can grow without quorum sensing, with the consequence that all QS-coupled properties (e.g., biofilm formation, virulence factors, etc.) will be inactivated causing relevant changes in the ecological functions of the bacterial population. Accordingly, the interference with the QS system by natural products is considered as very relevant for ecological interaction [83]. The Gram-negative bacterium *Ps. aeruginosa* is an opportunistic human pathogen that exhibits various antibiotic resistance mechanisms and virulence factors, such as biofilm formation. Given the limited antimicrobial arsenal against the resistant strains of this bacterium, the discovery of new active compounds against *Ps. aeruginosa* is a priority [84]. *Pseudomonas* species are also widely distributed in marine systems. Hence, activity in this assay can be an indication for activity relevant for bacterial biofilm fouling of marine surfaces. 

In general, nearly all extracts of static and shaken PC and AS cultures of *Z. marina* associated fungi showed potent QSI activity with poor inhibition against *A. fischeri*. The only exceptions were the AS and AS-sh extracts of strain 8 and the AS extract of strain 2. In contrast, many static and shaken CA, WH, and GPY extracts poorly inhibited QS and the growth of *A. fischeri*. It is noteworthy that all static and shaken PC and AS crude extracts (except for strain 8) contained the metabolite **10** (patulin). Besides being a mycotoxin, patulin, isolated from a *P. coprobium*, was found to inhibit quorum sensing in *Ps. aeruginosa* [85]. Therefore, this compound might have had some impact on the QSI activity. However, as the crude extracts of the non-*Penicillium* strains lacked compound **10**, some other QSI metabolites might also be present. Extracts from static and shaken PC and AS cultures of almost all *Penicillium* strains strongly inhibited the growth of *V. anguillarum* and *Ps. elyakovii*, except those from strain 8. Patulin (**10**) has been also reported to exhibit antimicrobial activity [86,87], which might explain the activity of the crude extracts containing **10**. In contrast to the *Penicillium* strains, none of the non-*Penicillium* strains inhibited the growth of *V. anguillarum* and *Ps. elyakovii*. Some *Penicillium* PC and AS culture extracts inhibited the growth of *Ps. aeruginosa,* although no specific biofilm formation inhibition was recorded. In contrast, the non-*Penicillium* strains failed to show any significant inhibition of biofilm formation or the growth of *Ps. aeruginosa*. In general, the bioassays showed that many *Z. marina* associated fungi (especially those grown in PC and AS media) were active against a variety of Gram-negative bacteria. Furthermore, compounds like patulin (**10**) and aflatoxin M1 (**54**) are known for being potent mycotoxins. Due to reduced chemical machinery and defense mechanisms of *Z. marina* during its re-adaptation from land to sea [4,8], these fungal SMs might have played a role in the chemical protection of this plant through inhibition against growth, QS, and formation of biofilm by marine pathogens. Furthermore, the observed bioactivities might help to generate new drugs against Gram-negative, clinically, and environmentally relevant pathogens in the future. 

In conclusion, we have isolated and identified, for the first time by a systematical approach, the cultivable fungal community associated with the Baltic *Z. marina*. The use of five different media and two culture regimes, as well as the application of a rapid dereplication method using HPLC-DAD-MS, enabled the identification of optimal culture conditions, revealing the chemical diversity and bioactivity of the fungal extracts. Because of their diverse SMPs with putatively new compounds and antimicrobial and/or QS inhibitory activities against Gram-negative bacteria, several strains, e.g., 1, 6, 8, and 11, will be submitted to large-scale cultivation followed by isolation and structure elucidation of their bioactive components. 

## 4. Materials and Methods 

### 4.1. Collection of Zostera marina

Fifteen living specimens of *Z. marina* were collected by SCUBA diving in a water depth of approx. 3 m in Friedrichsort (54°23′N; 10°11′E) around Kiel Fjord (Baltic Sea, Germany). The eelgrass leaf specimens were stored in sterile plastic bags at 4 °C before being transported and processed in the laboratory. For sampling the sediment surrounding the roots of *Z. marina* plants, 50 mL sterile Falcon tubes were used for coring the uppermost sediment surrounding the roots (five sediment samples collected). All samples were placed into a cool box, transported to the laboratory, and processed immediately.

### 4.2. Isolation of Fungi

The *Zostera* leaf specimens were cut (0.5 cm) and placed into a sterile innuSPEED Lysis Tube (Analytik Jena AG, Jena, Germany) together with 100 µL of sterile saline and homogenized for 6 min at a frequency of 30 s^−1^ in a RetschMM200 mixer mill (Retsch GmbH, Haan, Germany). Homogenates of *Z. marina* leaf subsamples and sediment samples were diluted 1:10 and 1:100 with sterile water for a dilution series. The 100 µL of each dilution step (10^−1^ and 10^−2^), 15 µL of undiluted homogenate, and a small spoon of sediment surrounding the roots were plated onto five Agar media; four Glucose-Yeast-Malt 4 medium (GYM4, glucose monohydrate 4 g, yeast extract 4 g, malt extract 4 g, CaCO_3_ 2 g, aqua dest. ad 1 L, pH 7.2), Glucose-Peptone-Yeast medium (GPY, glucose monohydrate 1 g, peptone 0.5 g, yeast extract 0.1 g, NaCl 15 g, agar 13 g, aqua dest. ad 1 L, pH 7.3), Marine broth medium (MB, Bacto, agar 15 g, aqua dest. ad 1 L), Tryptic Soy Broth 12 medium (TSB12, 12 g tryptic soy broth, 5 g NaCl, agar 15 g, aqua dest. ad 1 L, pH 7.2), and Hastings medium (HS, Na_2_HPO_4_x 12H_2_O 9.35 g, KH_2_PO_4_ 1 g, (NH_4_)_2_SO_4_ 0.5 g, MgSO_4_ × 7H_2_O 0.21 g, NaCl 30 g, trypton 5 g, yeast extract 3 g, glycerol 2 mL, agar 18 g, aqua dest ad 1 L, pH 7.4), for the cultivation of associated microbes. The inoculated Petri dishes were stored at 22 °C in the dark. All plates were checked for different morphotypes. Different morphotypes were isolated by streaking on new agar plates of the corresponding media to obtain axenic cultures, whenever they occurred in a total time frame of 21 days.

### 4.3. Identification of Fungal Strains

Fungal morphology and ITS sequences were used for taxonomic identification of *Z. marina* associated fungi. Fungal strains were studied by macroscopic (colony morphology) and microscopic means (hyphae, conidia, conidiophors, septation). Molecular identification was based on the sequence of the ITS1 nrDNA region. Fungal genomic DNA was obtained by simple cell lysis in nuclease-free water with a Retsch mill model MM200 (Retsch GmbH, Haan, Germany). 1 µL of this extract was used as a template for PCR amplifying ITS1-5.8S-ITS2 region with the primers [88] in a total reaction volume of 25 µL (12.5 µL Dream Taq Master Mix (ThermoFisher Scientific, Waltham, MA, USA), 1 µL of each primer (concentration 10 μM, ITS1 [5′-TCC GTA GGT GAA CCT GCG G-3′] and ITS4 [5′-TCC TCC GCT TAT TGA TAT GC-3′] [88]), and 9.5 µL of DNA free water (ThermoFisher Scientific, Waltham, MA, USA). PCR was performed in a T-1 thermocycler (Biometra, Göttingen, Germany) using the following conditions for amplification: denaturation 94 °C for 8 min, 35 cycles of denaturation 94 °C for 30 s, annealing 48 °C for 45 s, elongation 72 °C for 3 min, final elongation 72 °C for 10 min, cooling 4 °C [88]. PCR products were checked for correct length by gel electrophoresis using an SYBR safe (ThermoFisher Scientific, Waltham, MA, USA) pre-stained 1% TBE Agarose gel and Gene ruler 1 Kb DNA ladder (Thermo ScientificGeneRuler™, Schwerte, Germany) as length control. Electrophoresis conditions were 120 V for 20 min. Positive PCR products were analyzed by Sanger sequencing at the Institute of Clinical Molecular Biology (Kiel University) using the primer ITS4. All sequences were processed via the software ChromasPro V1.33 (technelysium Pty Ltd., Brisbane, Australia) and compared to the NCBI database (https://www.ncbi.nlm.nih.gov/genbank/) using the BLAST algorithm. 

### 4.4. Cultivation and Extraction of Marine Fungi

Pre-cultures of fungi were cultivated on the same medium used for isolation of the respective strain for two weeks at 22 °C in the dark. Approximately 1 cm^2^ piece of overgrown agar was used to inoculate five liquid media (100 mL), including Potato-Carrot (PC, carrot 20 g, potato 20 g, aqua dest. ad 1 L, pH 7.2), Casamino Acids (CA, casein hydrolysate 30 g, yeast extract 4 g, K_2_HPO_4_ 0.5 g, sea salt mixture (Instant Ocean) 15 g, aqua dest. ad 1 L, pH 7.4), Wickerham15 (WH, glucose monohydrate 10 g, peptone from soymeal 5 g, yeast extract 3 g, malt extract 3 g, sea salt mixture (Instant Ocean) 15 g, aqua dest. ad 1L, pH 7.3), Glucose-Peptone-Yeast (GPY, glucose monohydrate 1 g, peptone 0.5 g, yeast extract 0.1 g, NaCl 15 g, aqua dest. ad 1 L, pH 7.3), Artificial Seawater (AS, glucose monohydrate 1 g, sea salt mixture (Instant Ocean) 15 g, aqua dest. ad 1 L, pH 7.5), for three weeks at 22 °C in the dark either in a shaking incubator (Edmund Bühler GmbH, Bodelshausen, Germany) with 120 rpm (abbreviated as “sh” cultures) or without shaking. The complete culture broth was homogenized at 19,000 rpm for 30 s using a T25 Ultra Turrax (IKA Werke GmbH & Co. KG, Staufen im Breisgau, Germany). The suspension was extracted with ethyl acetate (EtOAc) and centrifuged (4700 rpm) for 10 min at 4 °C. The aqueous phases were discharged, while the respective organic phase was evaporated to dryness at 40 °C by a rotary evaporator. The dried extracts were stored at –20 °C until work-up.

### 4.5. LC-DAD-MS Analyses and Dereplication

The crude extracts were analyzed by an HPLC (LaChrom Elite, VWR-Hitachi, VWR International, Radnor, PA, USA) system hyphenated to a Diode Array Detector L-2450 (DAD) and an Esquire 4000 Bruker Daltonics mass spectrometer (ESIMS). The extracts were dissolved in HPLC grade MeOH (1 mg/mL), filtered through a 0.2 µm PTFE syringe filter, and injected (20 µL) into the HPLC-DAD-MS system. Chromatographic separations were achieved at 40 °C using a monolithic C18 column (Onyx monolithic C18, 100 × 3.0 mm, Phenomenex, Torrance, CA, USA). The mobile phase used was A: 95.0% MilliQ-water/0.1% formic acid (Fluka) and B: ACN (Actu-All Chemicals BV)/0.1% formic acid, pumped at a rate of 2.0 mL/min with the following gradient: initial, 95% A; 0–4 min 40% A; 6–6.9 min 0% A; 11.5–12.5 min 0% A, and a column reconditioning phase until 8.5 min at a flow of 2.5 mL/min. If not stated otherwise, measurements of crude extracts were conducted at positive ionization mode. As a result, monomer ions ([M + H]^+^, [M + Na]^+^) were primarily formed with dimer ions ([2M + H])^+^, [2M + Na]^+^) or potassium adducts ([M + K]^+^) being less commonly found. Both UV-VIS (200–500 nm) and MS data were evaluated using the programs Data Analysis and HyStar Post Processing (Bruker Daltonics, Bruker Corporation, MA, USA). For identification of known compounds, datasets of each crude extract were compared with entries in the database “Dictionary of Natural Products” as well as other publicly available databanks. Data were reconciled with database entries in matters of UV maxima, accurate mass, and biological source.

### 4.6. Bioassays 

All bioassays were performed in a 96-well microtiter format. The crude extracts were dissolved in DMSO (10 mg/mL stock solution) and diluted with the respective media to a final concentration of 0.5% DSMO in test volumes of 200 µL and extract concentrations of 100 µg/mL. Antibiotics (see below) served as positive controls, whereas inoculated medium (containing DMSO but no extracts), blanks (just medium), and DMSO served as negative controls. Assays were prepared in duplicates. 

#### 4.6.1. Antibacterial Activity

Crude extracts were screened for their antibacterial activity against *Ps. elyakovii* and *V. anguillarum* using resazurin as an indicator. Overnight cultures (28 °C, 160 rpm, Tropic Marine (TM, peptone 5.00 g, yeast extract 1.00 g, sea salt mixture (Instant Ocean) 30.00 g, aqua dest. ad 1 L, pH 7.6) of *Ps. elyakovii* were diluted to an optical density (OD) of 0.03 with medium, whereas those of *V. anguillarum* were diluted to an OD of 0.01 and inoculated into the test wells. Chloramphenicol was used as a positive control. Microplates were incubated at 28 °C and 200 rpm for 7 h (*Ps. elyakovii*) and 5 h (*V. anguillarum*). Subsequently, 10 µL of resazurin (0.1 mg/mL) solution was added to each well. Microplates were incubated again at RT and 200 rpm in the dark for 20 min. (*Ps. elyakovii*) and for 15 min. (*V. anguillarum*), and the fluorescence was measured at 590 nm using an excitation wavelength of 560 nm. 

#### 4.6.2. Quorum Sensing Inhibition

Pre-cultures (overnight at 28 °C and 160 rpm in HS medium) of *A. fischeri* were diluted to an OD of 0.01. Microplates were prepared as above, using 5-(bromomethylene)-2H(5H)-furanone and chloramphenicol as positive controls. Initial cell density was measured at 600 nm. The content of the microplate was transferred into a white microplate suitable for luminescence measurement and incubated at 28 °C and 200 rpm for 5 h. Subsequently, luminescence was measured in an Infinite M200 reader (TECAN Deutschland GmbH, Crailsheim, Germany) for 1000 ms. At a signal intensity of 1 × 10^6^ RLU (relative light unit) in the negative control, cell density was measured at 600 nm in a transparent microplate. 

#### 4.6.3. Biofilm Inhibition

TSB12 medium (40 mL) was inoculated with *Ps. aeruginosa* and incubated for 5 h at 37 °C and 200 rpm. This culture was diluted to an OD of 0.01 and incubated overnight at 37 °C and 200 rpm. For testing biofilm formation, this culture was dispersed into microtiter plates at a volume of 150 µL/well. Polymyxin B served as a positive control. A sterile PCR plate was submerged into the microplates, serving a surface for the biofilm. The constructs consisting of microplates and PCR plates were tightly fixed and incubated for 24 h at 37 °C. After incubation, PCR plates were washed three times with 0.9% sterile saline solution and subsequently dried at 60 °C for 1 h for fixation of biofilms. In the next step, each dried PCR plate was incubated in a 0.1% crystal violet solution at RT for 20 min. All plates were washed again three times with 0.9% sterile saline solution to remove unbound crystal violet. Finally, each PCR plate was placed into a new microplate prefilled with 200 µL 96% EtOH, and both were shaken at 22 °C and 100 rpm for 20 min. In the end, PCR plates were removed, and each microplate was measured for absorbance at 590 nm. As all crystal violet was originally bound to the biofilm, the amount of crystal violet estimated from the absorbance could be related to the amount of formed biofilm.

### 4.7. Statistics

All statistics were performed, using the soft wares Microsoft Office Excel 2016 and Past 3.10. Multivariate data analyses in the form of UPGMA (Unweighted Pair Group Method with Arithmetic mean) clustering were used for the determination of relatedness between different SMs of all (*Penicillium*) isolates per medium. UPGMA clustering was based on the calculation of Bray-Curtis dissimilarity. UPGMA clustering was based on the presence-absence matrices of the respective SMs. Only peaks with absorbance and mass information were counted as “present”. If this was not the case, the compound was declared as “absent”. For the construction of matrices used for UPGMA clusters, presence-absence data were normalized using the Hellinger transformation. This transformation was done for giving less weight to rare SMs in the transformed presence-absence table [89].

## Figures and Tables

**Table 1:** Identification of fungal strains isolated from the Baltic *Z. marina* sample based on morphological criteria as well as BLAST searches in GenBank of the Internal Transcribed Spacer (ITS) region. Closest relatives to fungal strains according to BLASTn are presented. L: leaf; S: sediment.

Strain Number	Origin	Morphology	Next Related Strain	Similarity (%)	GenBank Accession Number
1	L	*Phoma sp.*	*Phoma macrostoma*	100	MN166393
2	S	*Cladosporium sp.*	*Cladosporium langeronii*	99	MN166394
3	S	*Trichoderma sp.*	*Trichoderma harzianum*	100	MN166395
4	S	*Penicillium sp.*	*Penicillium antarcticum*	99	MN172369
5	S	*Penicillium sp.*	*Penicillium antarcticum*	100	MN166396
6	S	*Penicillium sp.*	*Penicillium antarcticum*	100	MN166397
7	S	*Penicillium sp.*	*Penicillium antarcticum*	100	MN166398
8	S	*Penicillium sp.*	*Penicillium atramentosum*	100	MN166399
9	S	*Penicillium sp.*	*Penicillium atrovenetum*	100	MN166400
10	S	*Penicillium sp.*	*Penicillium atrovenetum*	100	MN166401
11	S	*Penicillium sp.*	*Penicillium atrovenetum*	100	MN166402
12	S	*Penicillium sp.*	*Penicillium atrovenetum*	100	MN166403
13	S	*Penicillium sp.*	*Penicillium atrovenetum*	100	MN166404

**Table 2:** Dereplication of extracts from strains 1 (*Ph. macrostoma*), 2 (*C. langeronii*), and 3 (*T. harzianum*). n.d.: not detectable, n.k.: not known, UP: unknown peak.

Peak/Compound	Medium	UVmax (nm)	*m*/*z* [M + H]^+^	Identity	Reference
**1**	PC, PC-sh, CA-sh, WH, WH-sh	220, 245, 292, 342	229.1	Trioxsalen	[19]
**2**	WH-sh	222, 279	277.2	NK-A 17E-233II	[20]
**3**	CA, CA-sh	221, 289, 310	211.1	Agistatin D	[21]
**4**	CA, CA-sh	205, 216, 222, 277	295.1	Orsellide D	[22]
**5**	WH	215, 306	311.3	Orsellide C	[22]
**6**	CA, WH	223, 275, 288	599.3	Cyl-2	[23]
**7**	CA	223, 280	316.1	Quinolinone B	[24]
**8**	CA	227, 269, 334	282.1	Harzianopyridone	[25]
**9**	PC, CA-sh, WH, WH-sh	223, 261, 271, 281	282.2	2′-Deoxyribofuranosylguanine; 2N-Me	[26]
**UP1**	PC-sh	221, 292	325.2	n.k.	
**UP2**	PC, PC-sh, WH	220, 275, 325, 360	273.1	n.k.	
**UP3**	PC, PC-sh, WH, WH-sh	218, 296, 364	289.1	n.k.	
**UP4**	CA-sh, WH-sh	232	279.3	n.k.	
**UP5**	WH	215, 303	327.3	n.k.	
**UP6**	WH	218, 297	312.2	n.k.	
**UP7**	WH	220, 266, 276, 287	393.1	n.k.	
**UP8**	WH	226	245.2	n.k.	
**UP9**	WH-sh	222, 280, 292, 305, 319	626.2	n.k.	

**Table 3:** Observed bioactivities (%) of crude extracts from non-*Penicillium* strains at 100 µg/mL concentration. Quorum sensing (QS) and biofilm inhibition were compared to growth inhibition of their associated microorganisms, *A. fischeri* and *Ps. aeruginosa,* separated by /. Positive control. A: 10 µM 5-(bromomethylene)-2H(5H)-furanone, B: 10 µM chloramphenicol, C: 10 µM polymyxin B.

Medium	Inhibition (%) of *A. fischeri* QS/Growth Inhibition			Inhibition (%) of *Ps. aeruginosa* Biofilm/Growth Inhibition		
	*Phoma macrostoma*	*Cladosporium langeronii*	*Trichoderma harzianum*	*Phoma macrostoma*	*Cladosporium langeronii*	*Trichoderma harzianum*
PC	100/0	100/31	94/70	5 /24	0/0	0/3
PC-sh	97/0	93/37	47/0	0/6	0/7	17/3
CA	99/0	95/0	90/0	0/16	0/5	0/3
CA-sh	0/0	30/0	81/0	0/2	0/1	0/2
AS	73/0	0/0	47/5	0/8	0/7	0/9
AS-sh	53/0	77/0	76/0	12/1	0/3	11/4
WH	100/17	98/0	97/46	0/21	0/3	0/1
WH-sh	80/0	95/0	70/0	10/5	0/1	0/20
GPY	34 /0	1/13	20/0	0/5	0/0	0/2
GPY-sh	7/29	40/0	0/35	4/3	0/1	0/9
Standard	100_A_/100_B_	100_A_/100_B_	100_A_/100_B_	100_C_/100_C_	100_C_/100_C_	100_C_/100_C_

## Supplementary Materials

The following are available online at https://www.mdpi.com/1660-3397/17/7/419/s1, Figures S1–S13: Metabolite profile of strains 1–13 cultured under static and shaking conditions in five different media; Figure S14: Chemical structures of dereplicated compounds in *Zostera marina* associated fungi; Table S1: Spectral data used for dereplication of *all Penicillium* spp. extracts.
