# Supplementary material for: Rapid Metabolome and Bioactivity Profiling of Fungi Associated with the Leaf and Rhizosphere of the Baltic Seagrass Zostera marina
Source: Mar Drugs. 2019 Jul 19;17(7):419. doi: 10.3390/md17070419 (PMC6669648; doi:10.3390/md17070419)
Supplement: Supplementary file 1 [file marinedrugs-17-00419-s001.pdf]

SUPPLEMENTARY MATERIALS

Rapid Metabolome and **Bioactivity** Profiling of Fungi Associated with Phyllosphere and Rhizosphere of Baltic Seagrass *Zostera marina*

Lars-Erik Petersen[1], Michael Marner[1], Antje Labes[1] and Deniz Tasdemir[1,2,*]

[1] GEOMAR Centre for Marine Biotechnology (GEOMAR-Biotech), Research Unit Marine Natural Products Chemistry, GEOMAR Helmholtz Centre for Ocean Research Kiel, Am Kiel-Kanal 44, 24106 Kiel, Germany; lars-erik.petersen@uni-oldenburg.de; michael.marner@ime.frauenhofer.de; antje.labes@hs-flensburg.de

[2] Faculty of Mathematics and Natural Sciences, Kiel University, Christian-Albrechts-Platz 4, 24118 Kiel, Germany

***** Correspondence: dtasdemir@geomar.de; Tel.: +49-431-600-4430; ORCID ID: 0000-0002-7841-6271

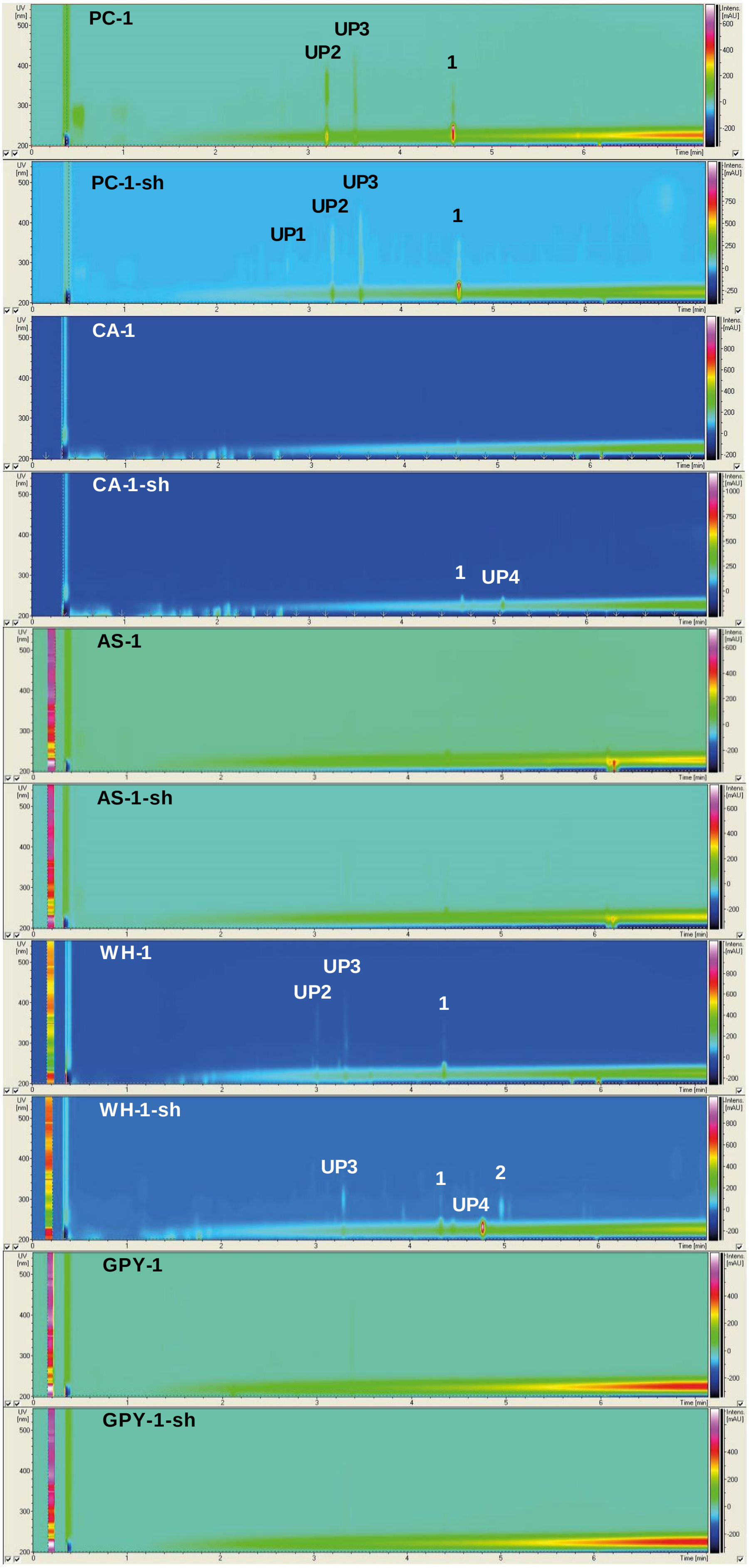

Figure S1. Metabolite profile of strain 1 (*Phoma macrostoma*) cultured under static and shaking (sh) conditions in five different media. Compounds are displayed as a function of UV absorption (nm) and intensity (mAU) vs. retention time (min). PC: potato-carrot medium, CA: casamino acids medium, AS: artificial seawater medium, WH: Wickerham's medium, GPY: glucose-peptone-yeast medium.

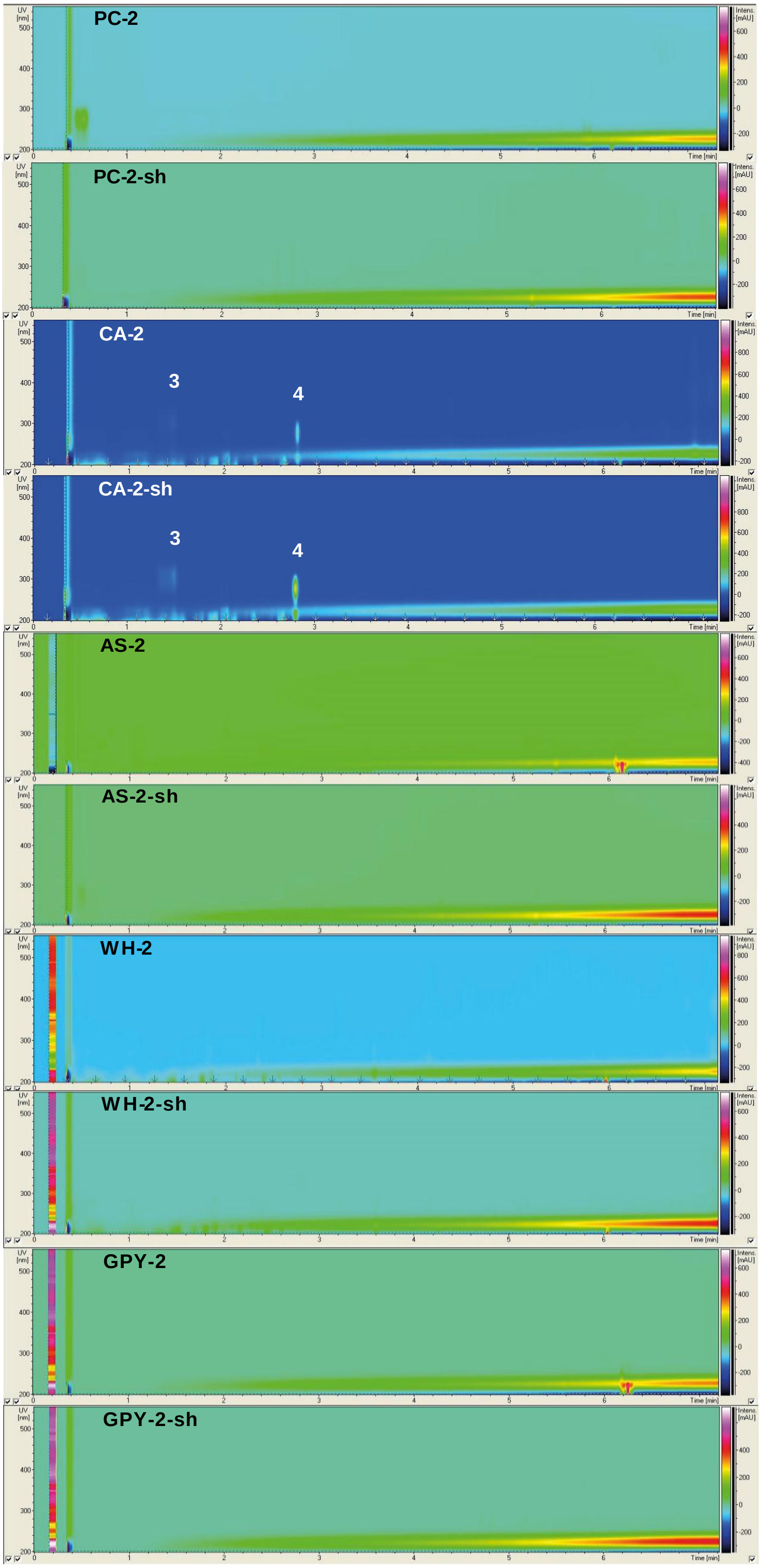

Figure S2. Metabolite profile of strain 2 (*Cladosporium langeronii*) cultured under static and shaking (sh) conditions in five different media.

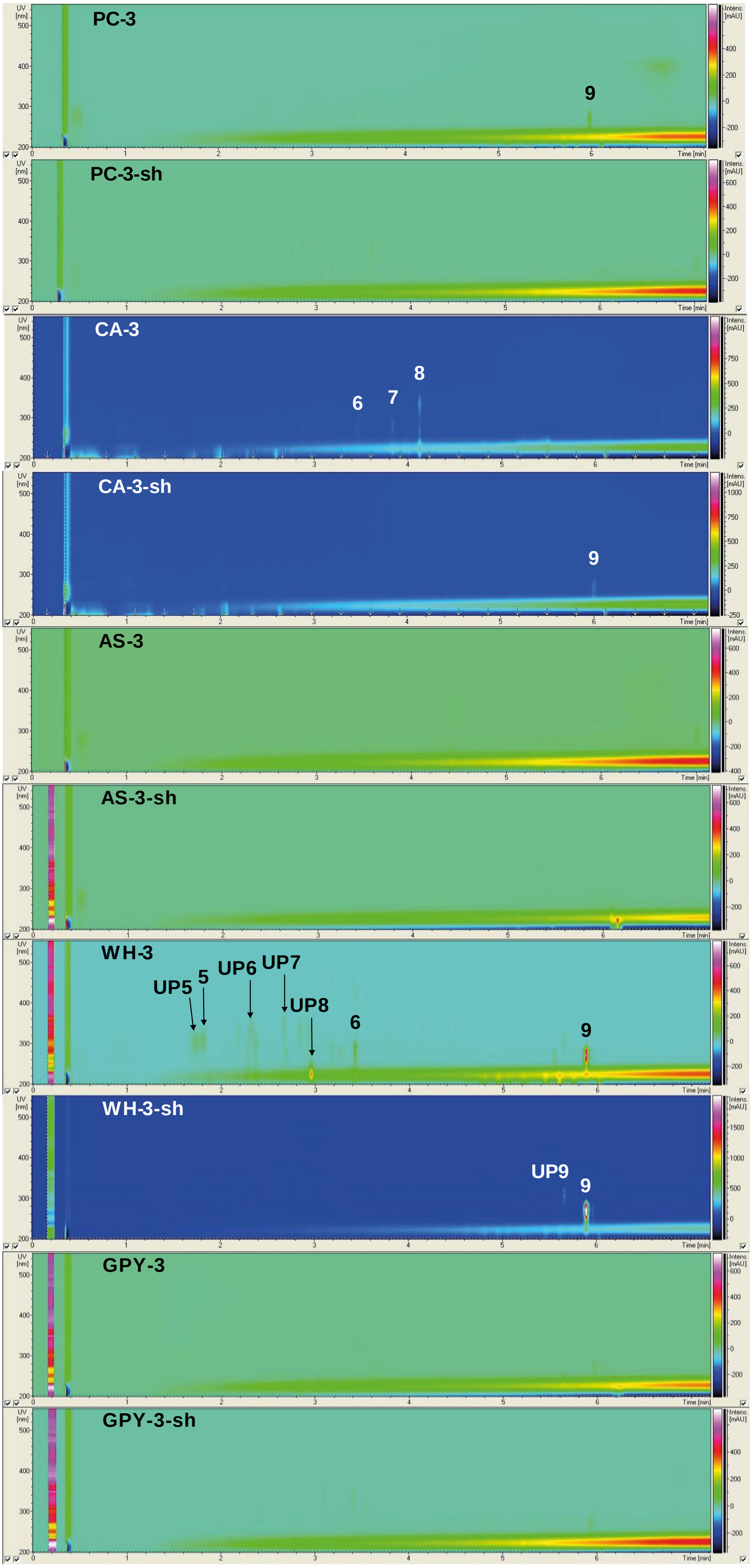

Figure S3. Metabolite profile of strain 3 (*Trichoderma harzianum*) cultured under static and shaking (sh) conditions in five different media.

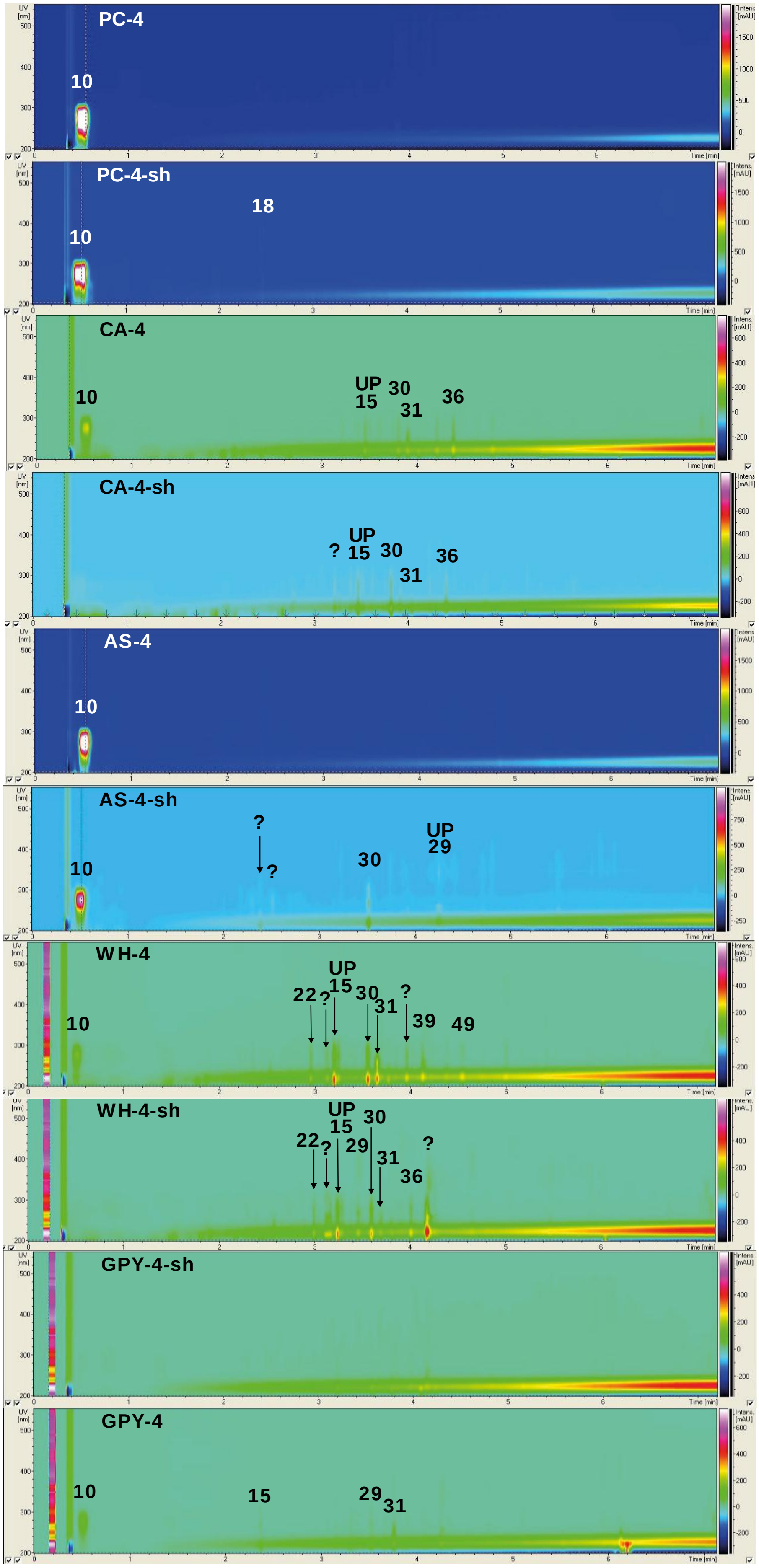

Figure S4. Metabolite profile of strain 4 (*Penicillium antarcticum*) cultured under static and shaking (sh) conditions in five different media.

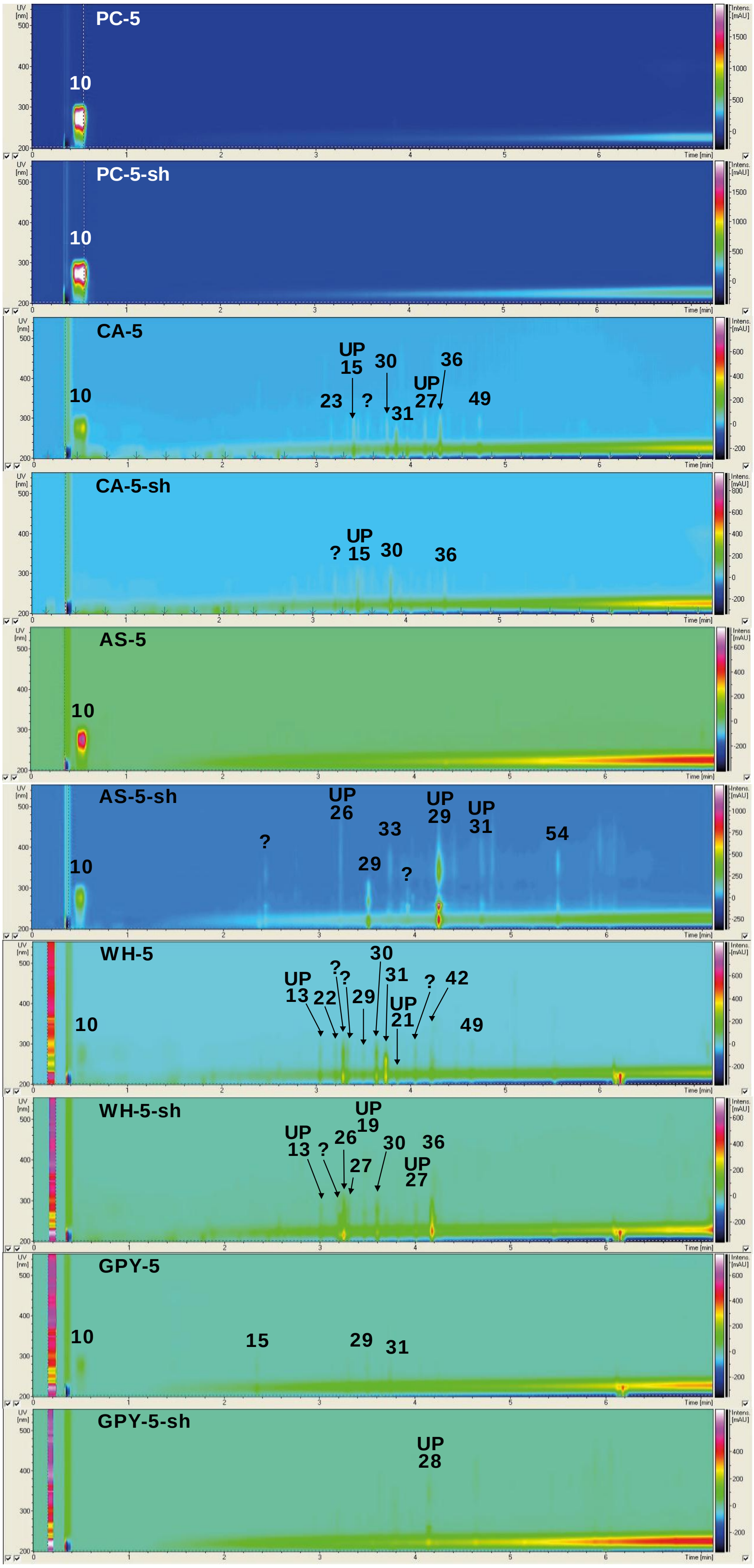

Figure S5. Metabolite profile of strain 5 (*P. antarcticum*) cultured under static and shaking (sh) conditions in five different media.

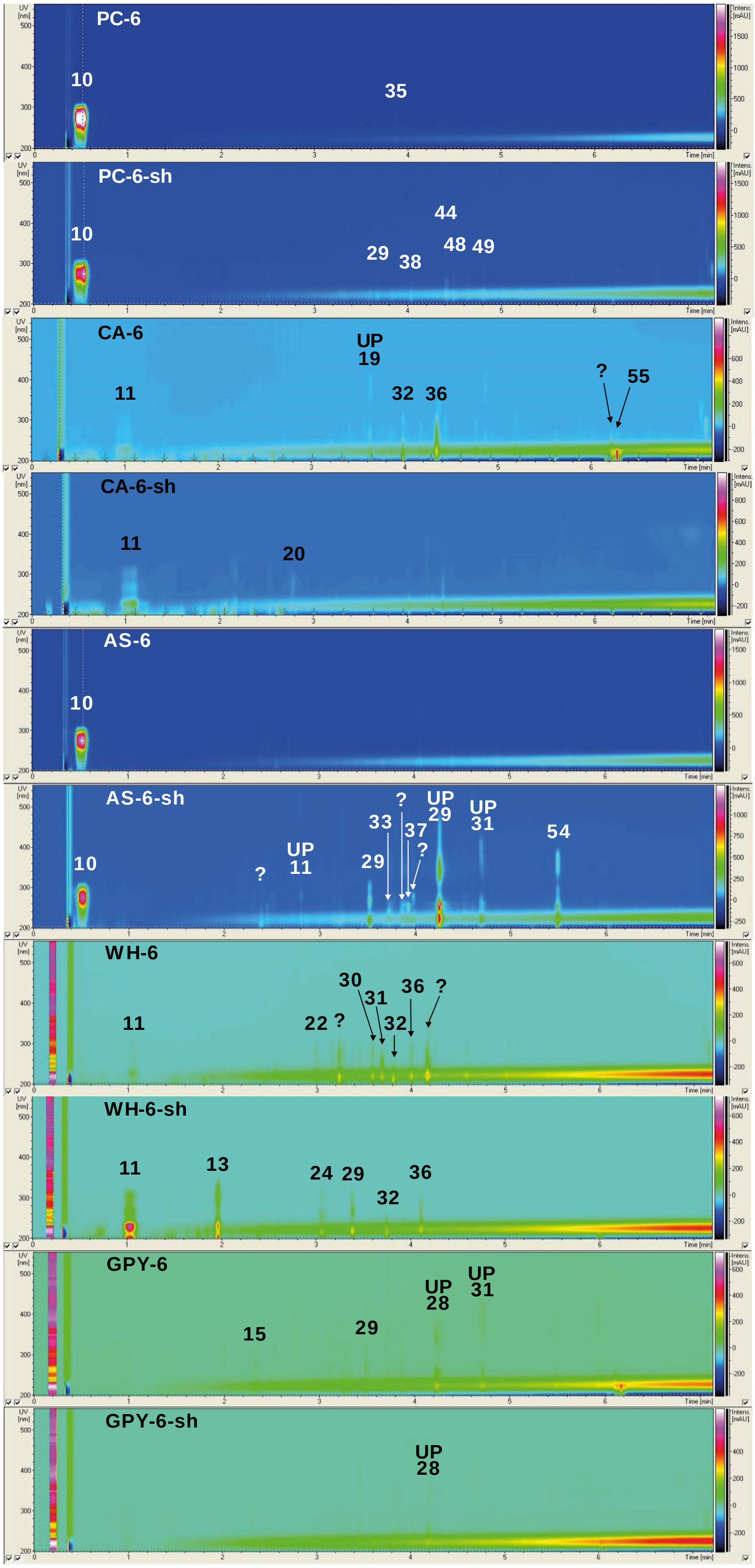

Figure S6. Metabolite profile of strain 6 (*P. antarcticum*) cultured under static and shaking (sh) conditions in five different media.

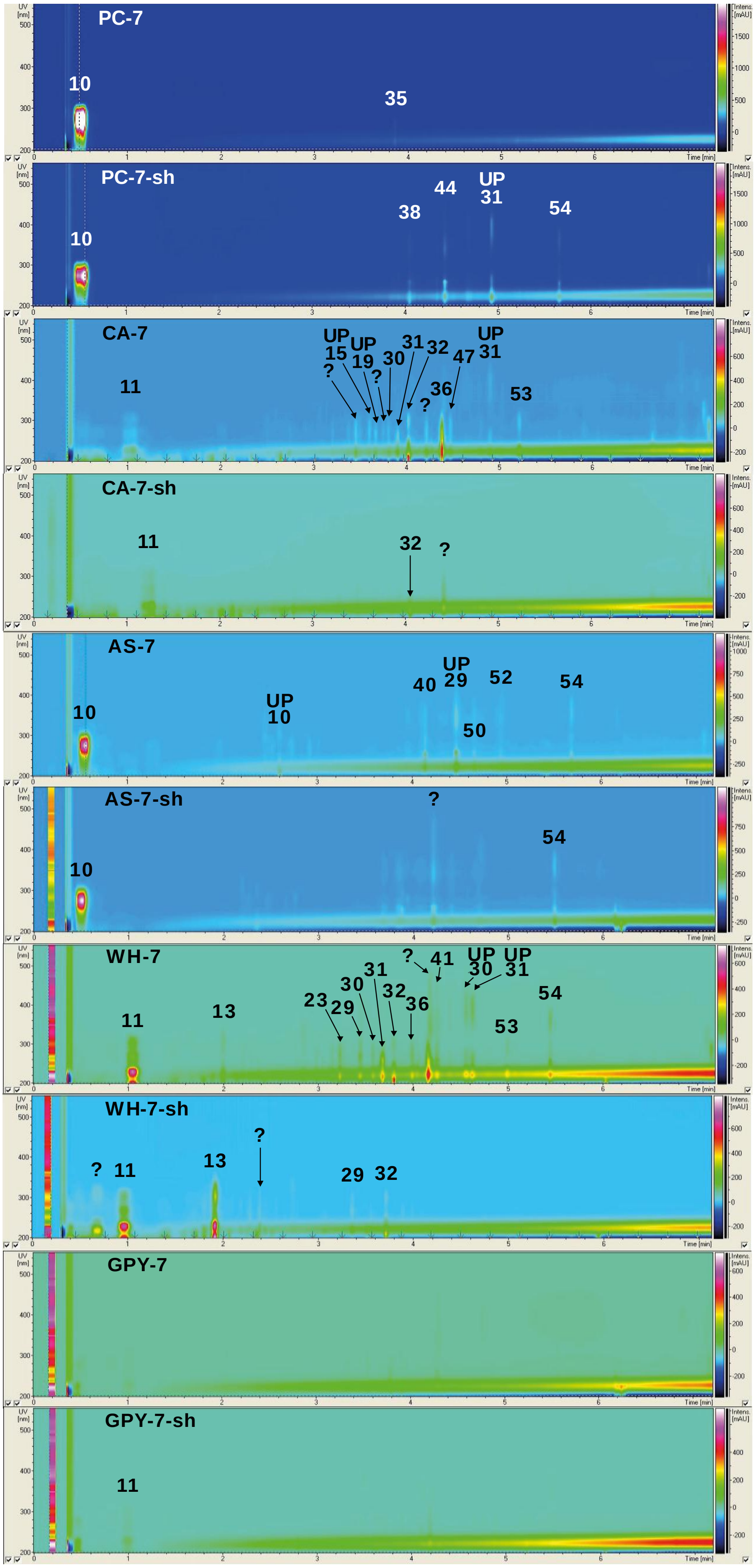

Figure S7. Metabolite profile of strain 7 (*P. antarcticum*) cultured under static and shaking (sh) conditions in five different media.

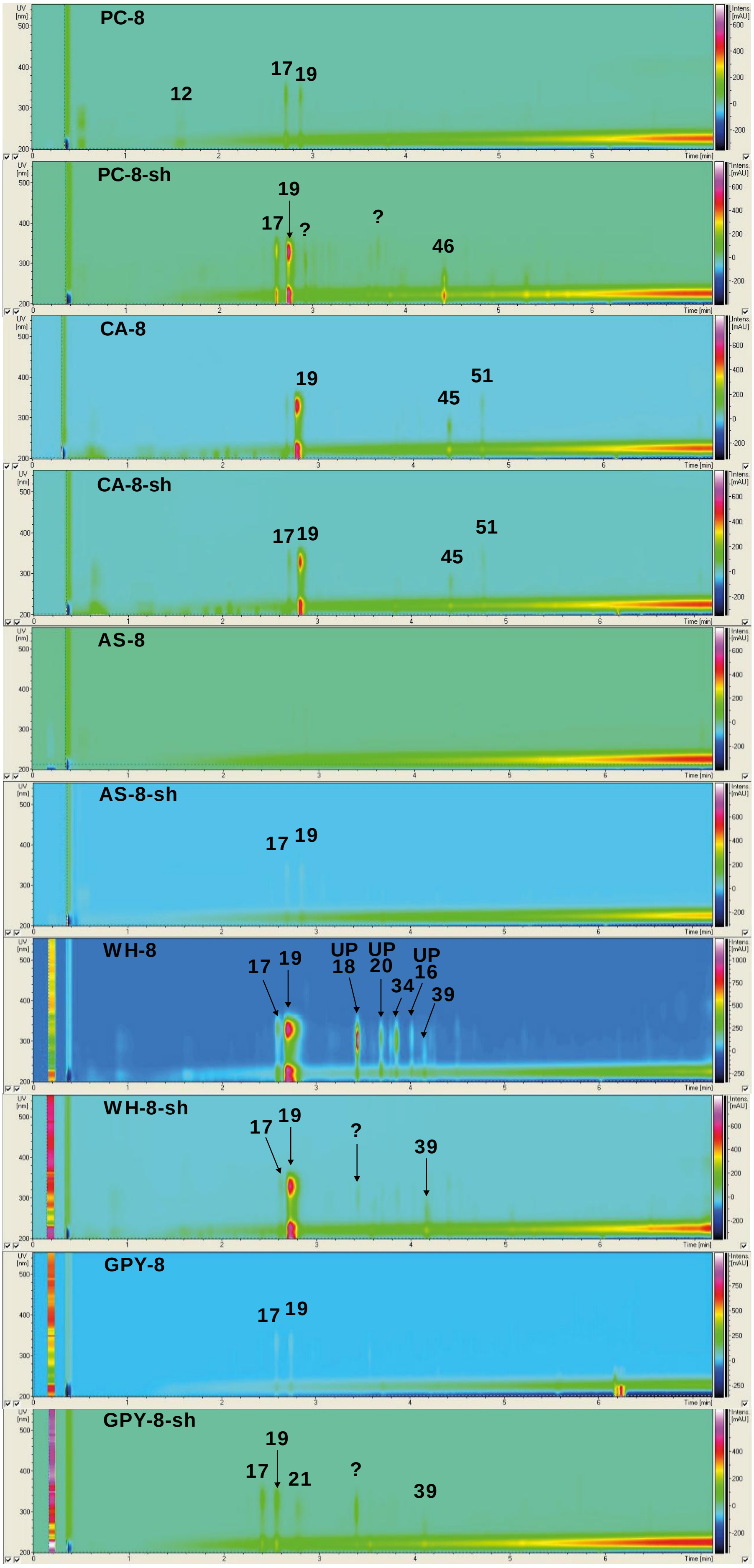

Figure S8. Metabolite profile of strain 8 (*P. atramentosum*) cultured under static and shaking (sh) conditions in five different media.

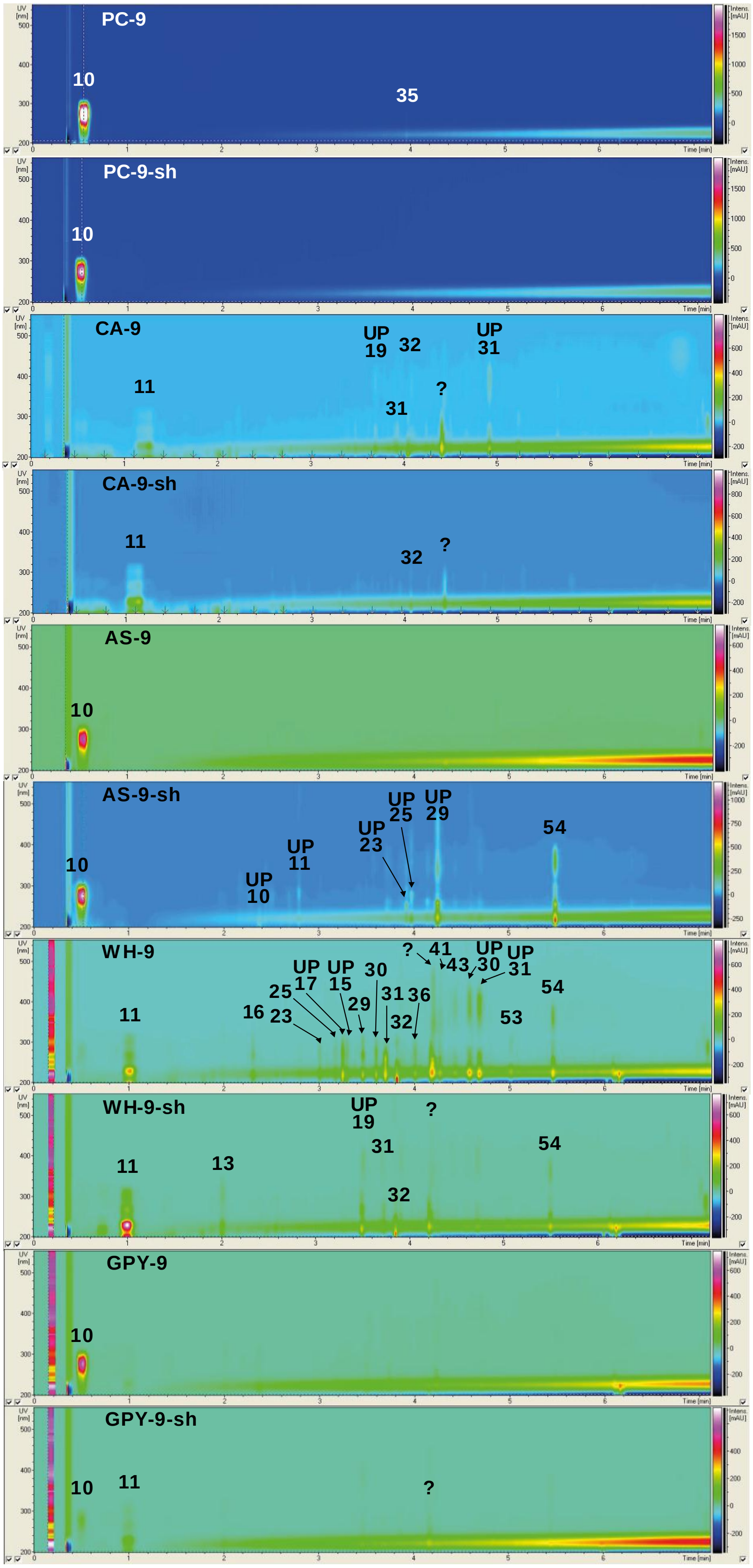

Figure S9. Metabolite profile of strain 9 (*P. atrovenetum*) cultured under static and shaking (sh) conditions in five different media.

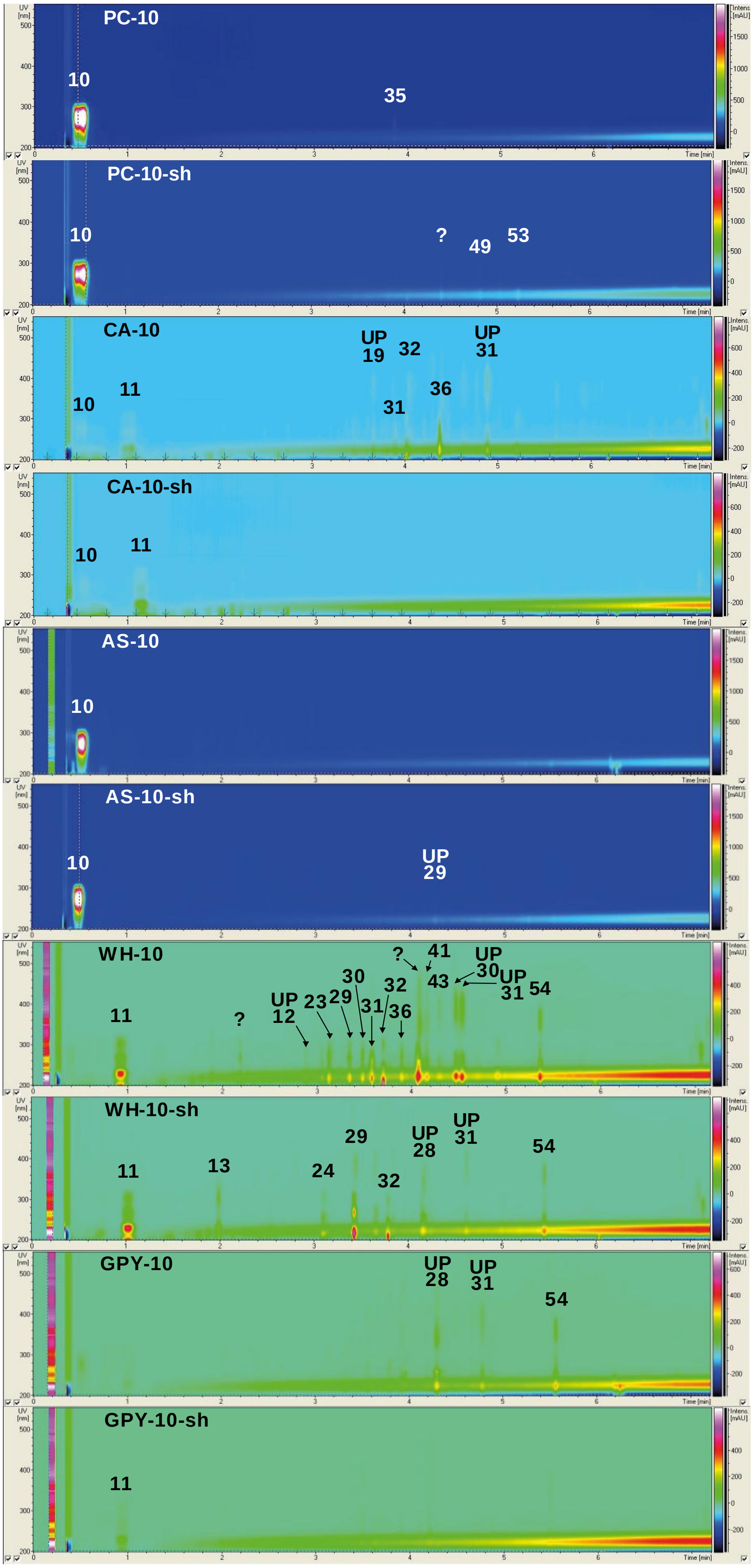

Figure S10. Metabolite profile of strain 10 (*P. atrovenetum*) cultured under static and shaking (sh) conditions in five different media.

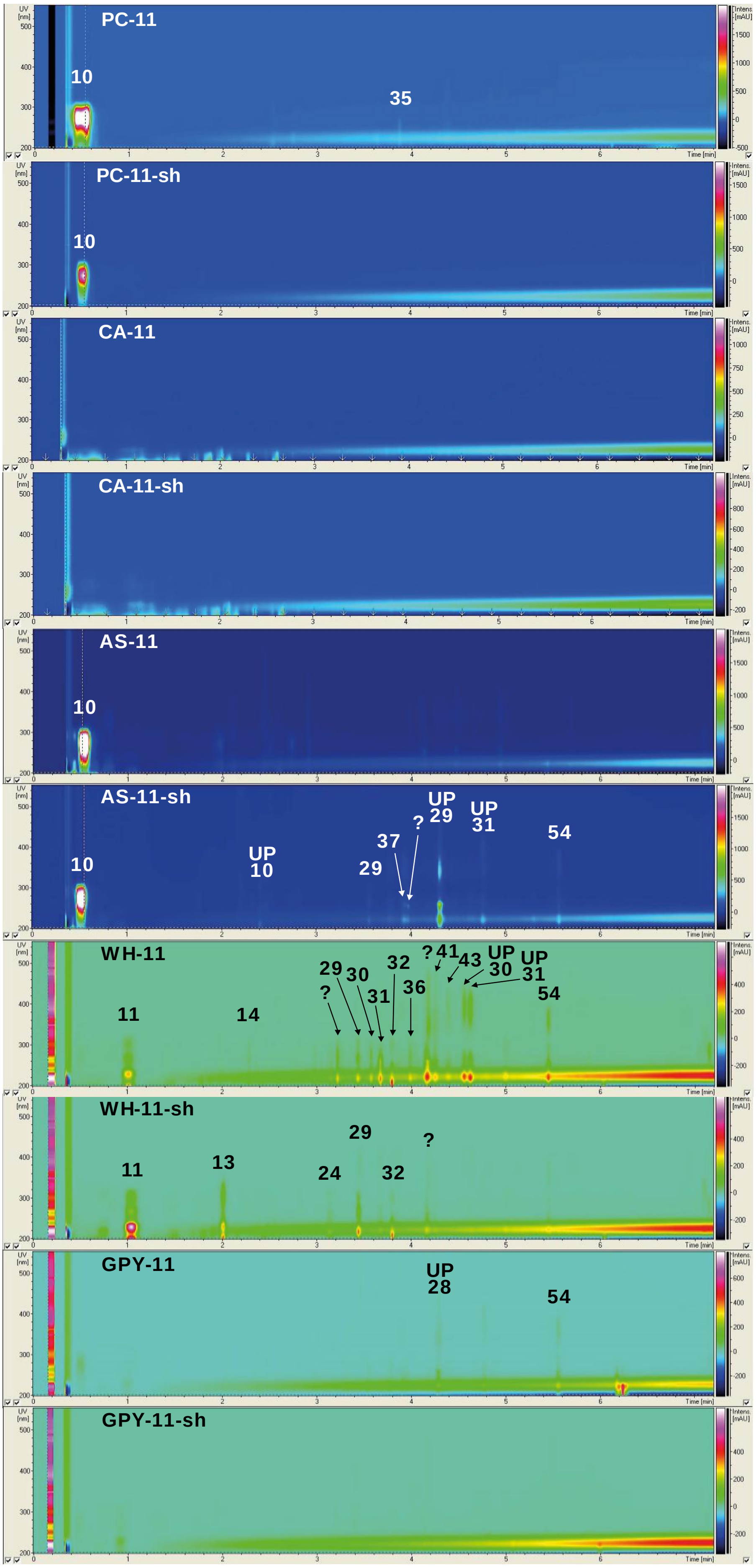

Figure S11. Metabolite profile of strain 11 (*P. atrovenetum*) cultured under static and shaking (sh) conditions in five different media.

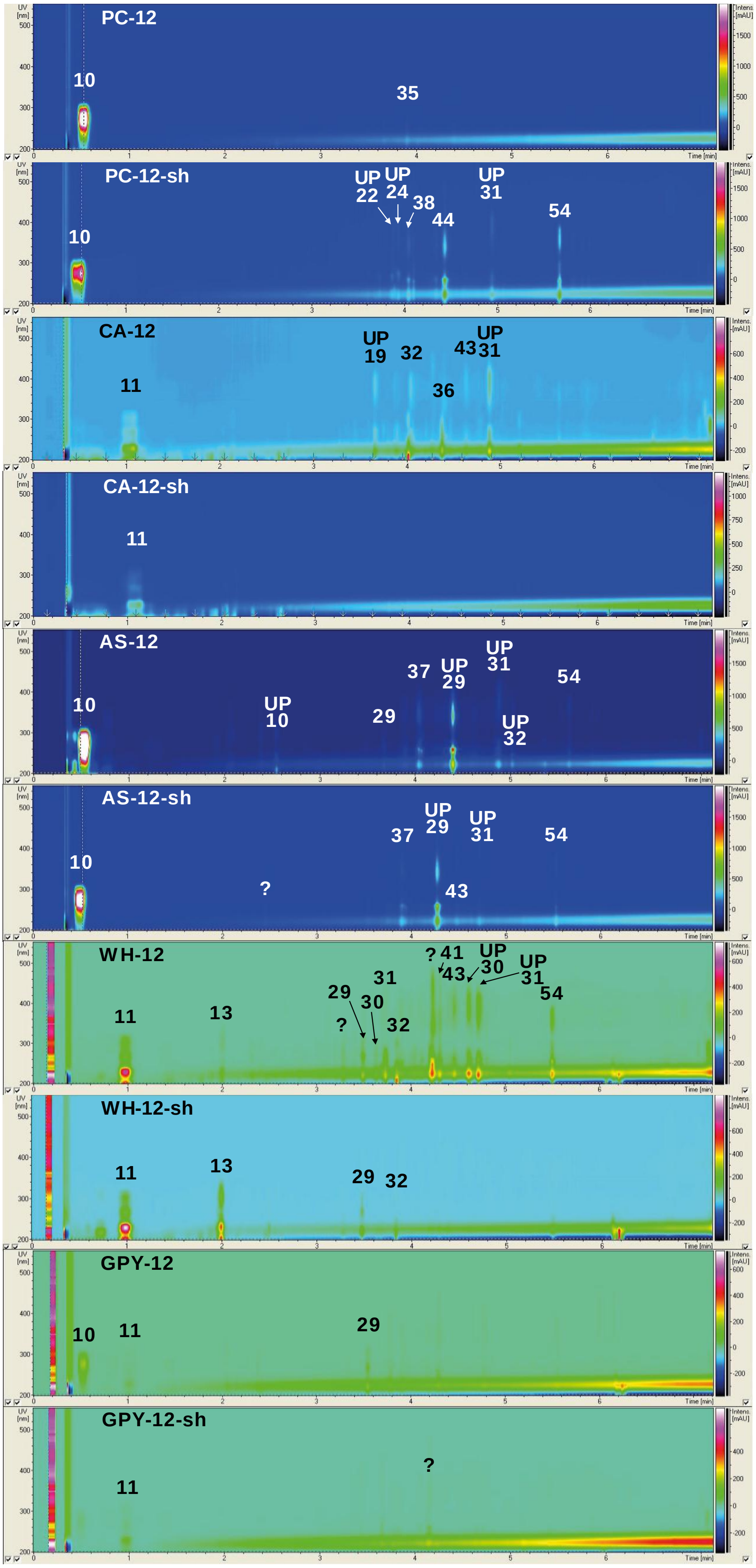

Figure S12. Metabolite profile of strain 12 (*P. atrovenetum*) cultured under static and shaking (sh) conditions in five different media.

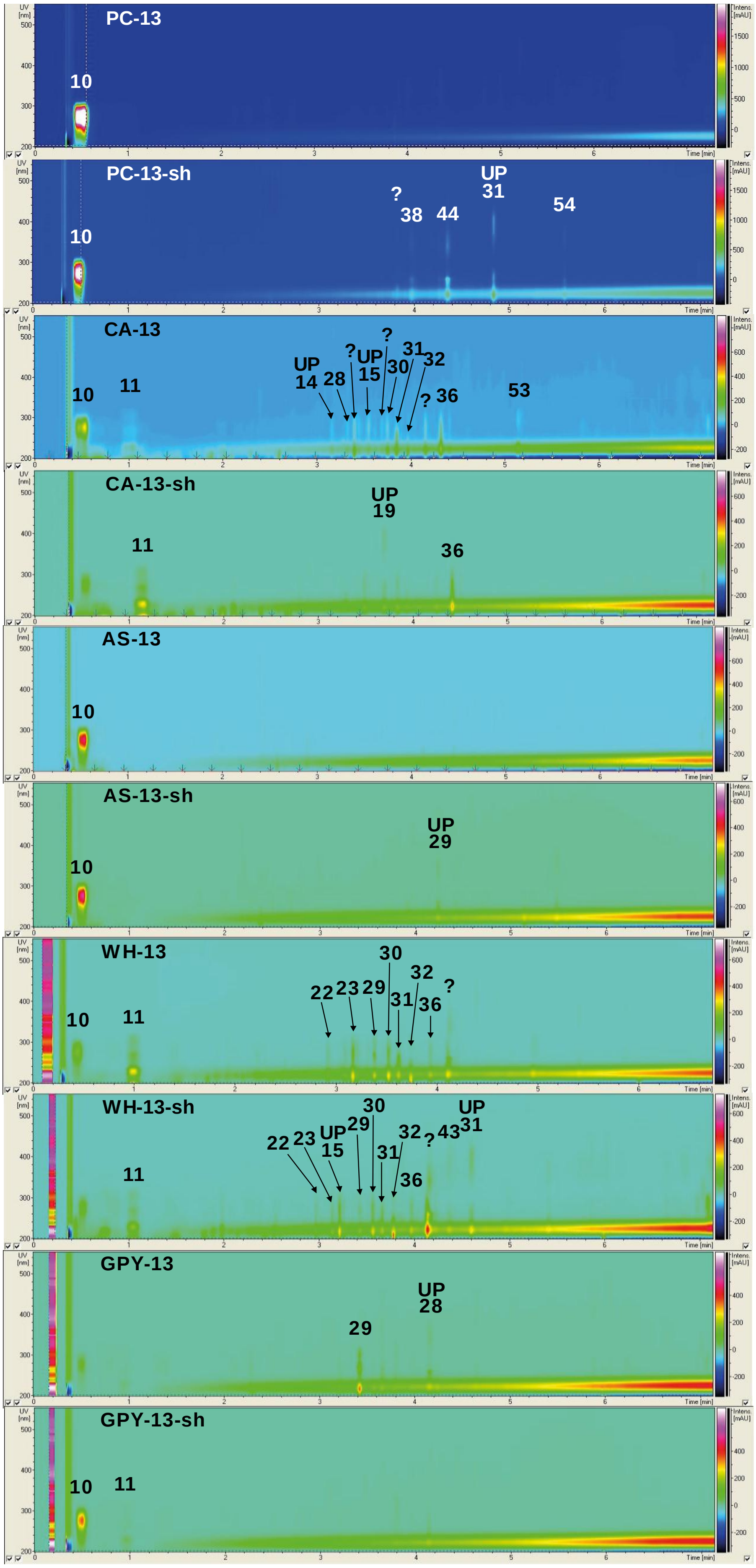

Figure S13. Metabolite profile of strain 13 (*P. atrovenetum*) cultured under static and shaking (sh) conditions in five different media.

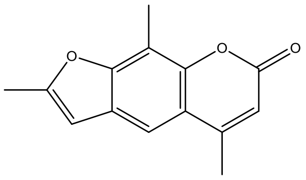

1

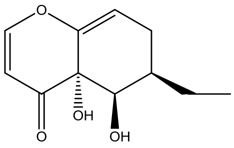

3

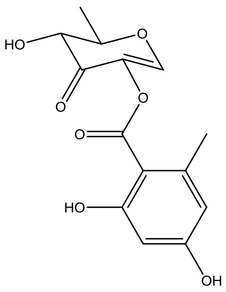

4

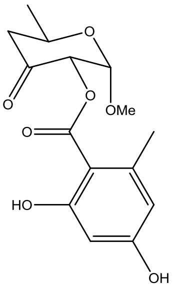

5

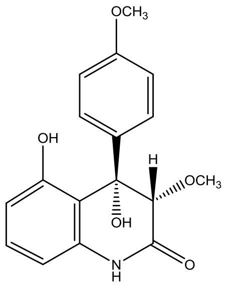

7

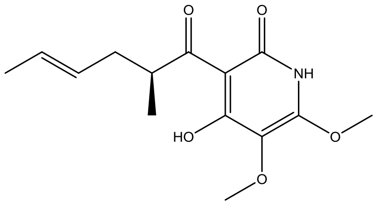

8

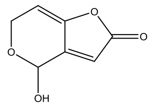

10

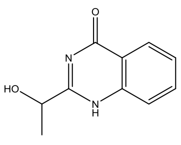

11

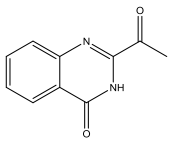

13

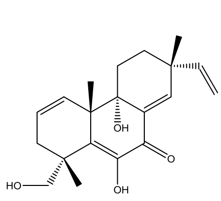

14

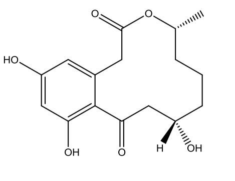

15

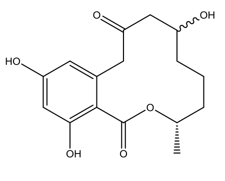

16

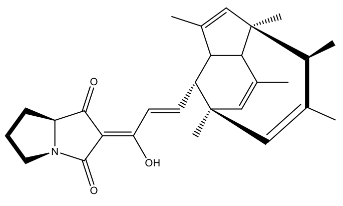

17

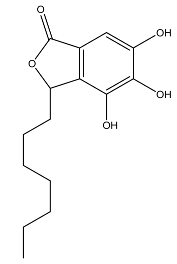

18

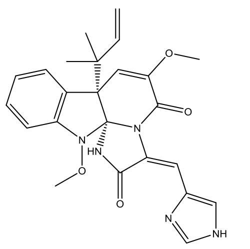

19

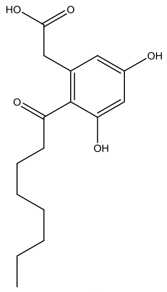

20

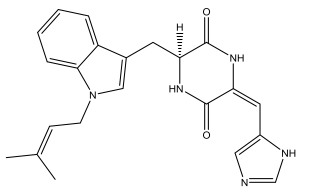

21

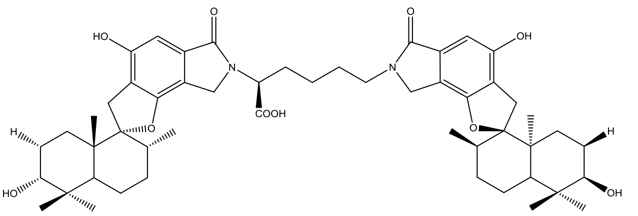

22

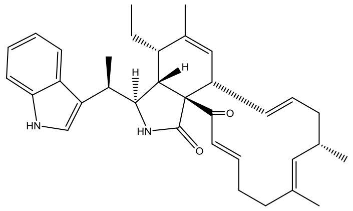

23

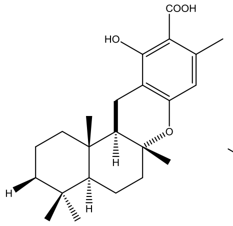

24

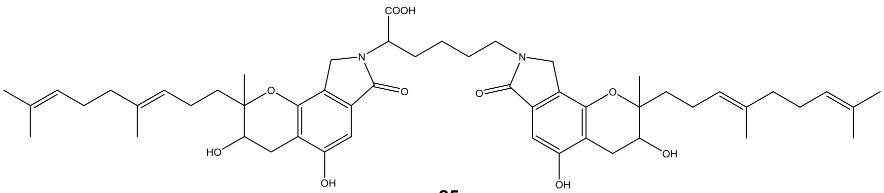

25

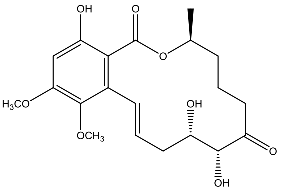

26

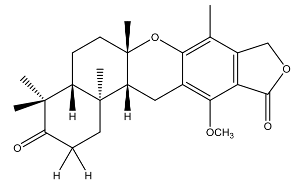

27

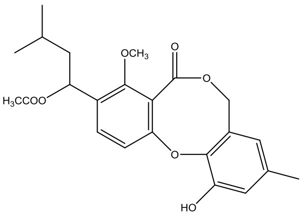

28

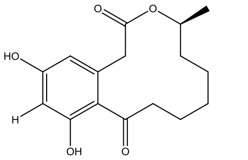

29

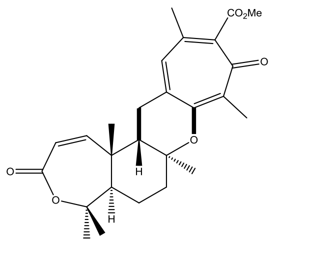

31

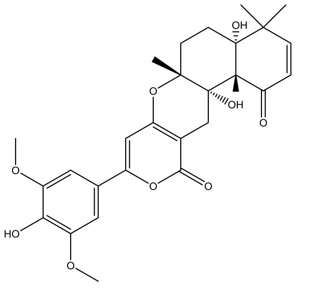

33

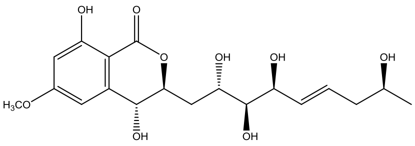

34

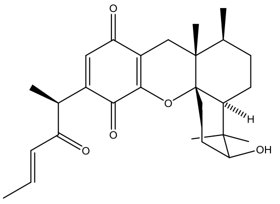

35

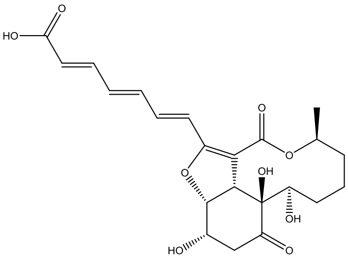

36

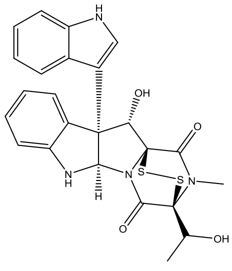

37

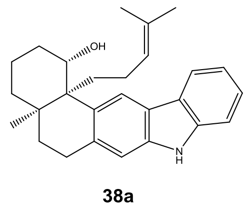

38a

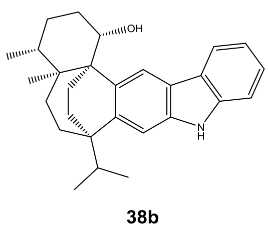

38b

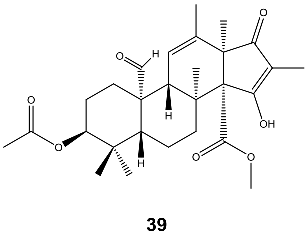

39

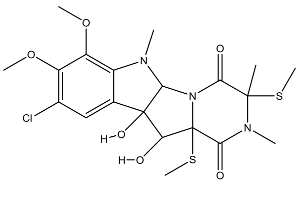

41

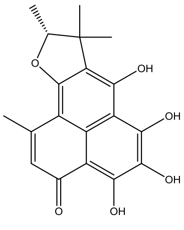

43

.

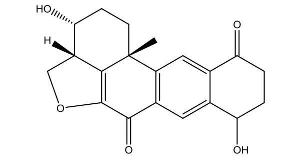

44

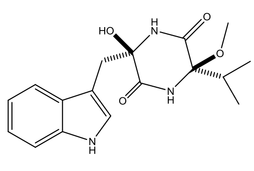

45

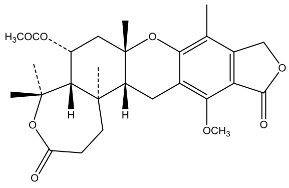

46

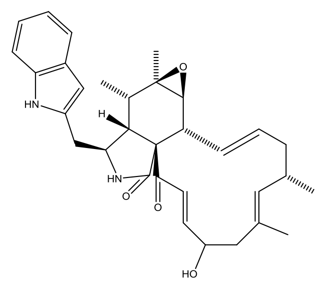

47

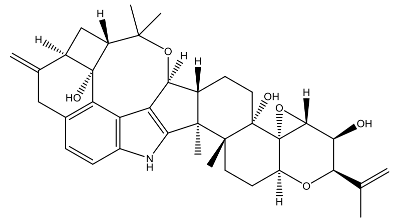

49

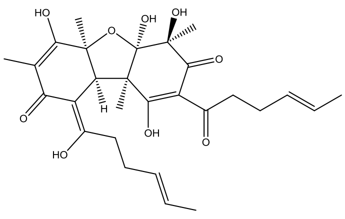

50

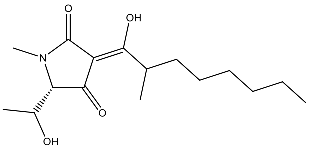

51a

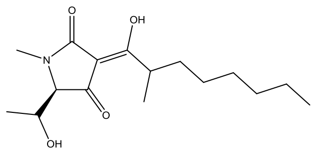

51b

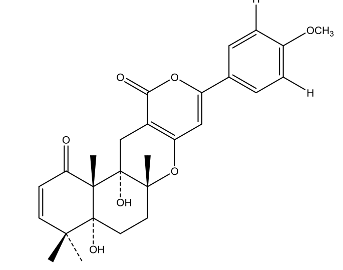

52

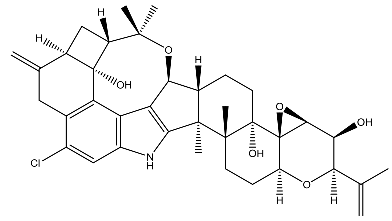

53a

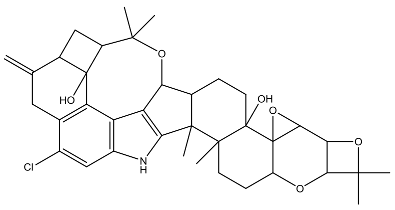

53b

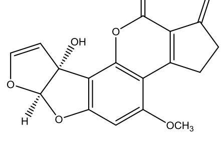

54

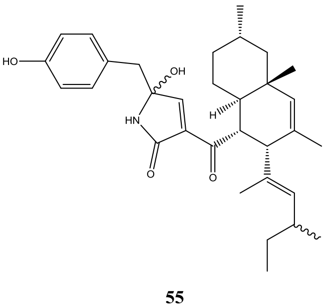

55

Figure S14. Chemical structures of dereplicated compounds in Table S1 (below). 38a: tubingensin A; 38b: tubingensin B; 51a: penicillenol A1; 51b: penicillenol A1 ; 53a: penitrem A ; 53b: pennigritrem

Table S1. Spectral data used for dereplication of all *Penicillium spp.* (strains 4-13) extracts. n.d.: not detectable, n.i.: not identified, UP: unknown peak.

Peak No.	~ UV_{max} (nm)	Mass $[H]^+$	Dereplication	Produced by strain	Produced in media
10	275	155.0	Patulin [1]	4, 5, 6, 7, 9, 10, 11, 12, 13	PC, PC-sh, CA, CA-sh, AS, AS-sh, WH, WH-sh, GPY, GPY-sh
11	228, 267, 292, 313	191.0	Chrysogine, (S)-form [2]	6, 7, 9, 10, 11, 12, 13	CA, CA-sh, WH, WH-sh, GPY, GPY-sh
12	215, 260, 298	269.1	Globosumone B [3]	8	PC
13	204, 230, 303	189.0	2-Acetyl-4(3H)-quinazolinone [4]	6, 7, 9, 10, 11, 12	WH, WH-sh
14	216, 267, 299	331.1	Libertellenone B [5]	11	WH
15	218, 267, 300	309.1	Curvularin, (S)-form, 11 α-Hydroxy [6]	4, 5, 6, 13	GPY
16	216, 268, 300	309.1	7,8-Dihydro-7α-hyroxyresorcylide [7]	9	WH
17	223, 330, 345	434.1	PF 1018 [8]	8	PC, PC-sh, AS-sh, WH, WH-sh, GPY, GPY-sh
18	220, 269, 350	281.0	Cytosporone E [9]	4	PC-sh
19	224, 279, 327, 342	448.2	Oxaline [10]	8	PC, PC-sh, CA, CA-sh, AS-sh, WH, WH-sh, GPY, GPY-sh
20	220, 276	295.1	Cytosporone A [9]	6	CA, CA-sh
21	220, 304	390.2	Tryhistatin [11]	8	GPY-sh

22	222, 273	883.4	Stachybocin A [12]	4, 5, 6, 13	WH, WH-sh
23	220, 270	511.3	Chaetoglobosin 510 [13]	5, 7, 9, 10, 13	CA, WH, WH-sh
24	215, 257, 305	373.1	Hongoquercin A [14]	6, 10, 11	WH-sh
25	221, 260, 279	883.4	Antibiotic SMTP 8 [15]	9	WH
26	215, 263, 279, 298	395.2	Hamigeromycin D [16]	5	WH-sh
27	220, 268, 280, 298	413.2	Austalide K [17]	5	WH-sh
28	221, 273, 368	415.3	Purpactin A [18]	13	CA
29	217, 267, 300	293.1	Curvularin, (S)-form [19]	4, 5, 6, 11, 12	AS, AS-sh, WH, WH-sh, GPY
30	220, 275	437.2	3-O-Methylterprenin	4, 5, 6, 7, 9, 10, 11, 12, 13	CA, CA-sh, WH, WH-sh
31	219, 259	441.2	Tropolactone C [20]	4, 5, 6, 7, 9, 10, 11, 12, 13	CA, CA-sh, WH, WH-sh, GPY
32	208, 220, 300	402.2	* see full name as footnote	6, 7, 9, 10, 11, 12, 13	CA, CA-sh, WH, WH-sh
33	221, 243, 261, 354	513.2	Territrem C [21]	5, 6	AS-sh
34	220, 300	399.2	Aigialomycin F [22]	8	WH
35	222, 258	441.2	Podosporin A [23]	6, 7, 9, 10, 11, 12	PC

36	220, 271	435.2	Dictyosphaeric acid B [24]	4, 5, 6, 7, 9, 10, 11, 12, 13	CA, CA-sh, WH, WH-sh
37	220, 256, 288, 340	495.1	Bionectin B [25]	6, 11, 12	AS, AS-sh
38	219, 259, 341	402.2	Tubingensin A or B [26, 27]	6, 7, 12, 13	PC-sh
39	220, 262, 290	487.2	Andrastin A [28]	4, 8	WH, WH-sh, GPY-sh
40	220, 255, 288, 338	647.1	Orbuticin [29]	7	AS
41	260, 295, 348, 375	504.1	Sporidesmin D [30]	7, 9, 10, 11, 12	WH
42	225, 258, 280, 340	487.2	Antibiotic UK 88051 [31]	5	WH
43	222, 260, 388	343.1	Atrovenetin [32]	9, 10, 11, 12, 13	CA, AS-sh, WH, WH-sh
44	222, 250, 259, 340	341.2	Tetrahydrohalenaquinone A [33]	6, 7, 12, 13	PC-sh
45	222, 279	332.2	Polanrazine E [34]	8	CA, CA-sh
46	220, 261	487.0	Austalide I [17]	8	PC-sh
47	223, 260, 288	515.3	20-Dihydroprotochaetoglobosin III [35]	7	CA
48	221, 261, 300	307.2	Antibiotic PO1	6	PC-sh
49	223, 288, 347	600.4	Penitrem E [36]	4, 5, 6, 10	PC-sh, CA, WH

50	222, 260, 340	517.1	2',2'',3',3''-Tetrahydrobisvertinolone [37]	7	AS
51	222, 269, 334	298.1	Penicillenol A_1 or A_2 [38]	8	CA, CA-sh
52	222, 259, 343	467.1	Arisugacin B [39]	7	AS
53	225, 296, 347	634.2	Penitrem A or Pennigritrem [40]	7, 9, 10, 13	PC-sh, CA
54	255, 297, 356, 380	329.1	Aflatoxin M1 [41]	5, 6, 7, 9, 10, 11, 12, 13	PC-sh, AS, AS-sh, WH, WH-sh, GPY
55	<200, 221, 267	506.6	Talaroconvolutin B [42]	6	CA
UP 10	210, 242, 305	135.0	n.h.	7, 9, 11, 12	AS, AS-sh
UP 11	220, 278, 286	317.1	n.h.	6, 9	AS-sh
UP 12	220, 270	535.3	n.h.	10	WH
UP 13	222, 272	883.4	n.h.	5	WH, WH-sh
UP 14	220, 271	472.2	n.h.	13	CA
UP 15	219, 272	453.2	n.h.	4, 5, 7, 9, 13	CA, CA-sh, WH, WH-sh
UP 16	223, 309, 361	365.2	n.h.	8	WH
UP 17	216, 274	395.2	n.h.	9	WH

UP 18	218, 300, 360	851.4	n.h.	8	WH
UP 19	220, 268, 355, 385	516.2	n.h.	5, 6, 7, 9, 10, 12, 13	CA, CA-sh, WH-sh
UP 20	224, 240, 308	383.3	n.h.	8	WH
UP 21	222, 297	402.2	n.h.	5	WH
UP 22	222, 267, 350	421.2	n.h.	12	PC-sh
UP 23	220, 255, 288, 340	313.1	n.h.	9	AS-sh
UP 24	221, 274, 355, 395	485.2	n.h.	12	PC-sh
UP 25	216, 277, 358	301.1	n.h.	9	AS-sh
UP 26	220, 291, 364, 425	365.2	n.h.	5	AS-sh
UP 27	222, 276	435.2	n.h.	5	CA, WH-sh
UP 28	222, 259, 340	341.1	n.h.	5, 6, 10, 11, 13	WH-sh, GPY, GPY-sh
UP 29	222, 248, 259, 340	739.1	n.h.	4, 5, 6, 7, 9, 10, 11, 12, 13	AS, AS-sh
UP 30	222, 263, 372, 405	504.1	n.h.	7, 9, 10, 11, 12	WH
UP 31	220, 260, 358, 404	357.2	n.h.	5, 6, 7, 9, 10, 11, 12, 13	PC-sh, CA, AS, AS-sh, WH, WH-sh, GPY

UP 32	222, 245, 278, 344	389.1	n.h.	12	AS

* 4-Hydroxy-1-methoxy-5-phenyl-3 (tetrahydro-6-(3-hydroxy-1 methylpropyl)-3,5-dimethyl-2H-pyran-2-yl)-2(1H)-pyridinone

Supplementary References

1. Monteillier, A.; Allard, P.-M.; Gindro, K.; Wolfender, J.-L.; Cuendet, M. Lung cancer chemopreventive activity of patulin isolated from *Penicillium vulpinum*. *Molecules* **2018**, *23*, 636, DOI: 10.3390/molecules23030636.

2. Hikino, H.; Nabetani, S.; Takemoto, T. Structure and biosynthesis of chrysogine, a metabolite of *Penicillium chrysogenum*. *Yakugaku Zasshi* **1973**, *93*, 619–623, DOI: 10.1248/yakushi1947.93.5_619.

3. Bashyal, B.P.; Kithsiri Wijeratne, E.M.; Faeth, S.H.; Leslie Gunatilaka, A.A. Globosumones A-C, cytotoxic orsellinic acid esters from the Sonoran Desert endophytic fungus *Chaetomium globosum*. *J. Nat. Prod.* **2005**, *68*, 724-728, DOI: 10.1021/np058014b.

4. Tsantrizos, Y.S.; Xu, X.-J.; Sauriol, F.; Hynes, R.C. Novel quinazolinones and enniatins from *Fusarium lateritium* Nees. *Can. J. Chem.* **1993**, *71*, 1362-1367, DOI: 10.1139/v93-176.

5. Oh, D.-C.; Jensen, P.R.; Kauffman, C.A.; Fenical, W. Libertellenones A–D: induction of cytotoxic diterpenoid biosynthesis by marine microbial competition. *Bioorg. Med. Chem.* **2005**, *13*, 5267-5273, DOI: 10.1016/j.bmc.2005.05.068.

6. Greve, H.; Schupp, P.J.; Eguereva, E.; Kehraus, S.; Kelter, G.; Maier, A.; Fiebig, H.-H.; König, G.M. Apralactone A and a new stereochemical class of curvularins from the marine fungus *Curvularia* sp. *Eur. J. Org. Chem.* **2008**, 5085–5092, DOI: 10.1002/ejoc.200800522.

7. Küppers, L.; Ebrahim, W.; El-Neketi, M.; Özkaya, F.C.; Mándi, A.; Kurtán, T.; Orfali, R.S.; Müller, W.E.G.; Hartmann, R.; Lin, W.; et al. Lactones from the sponge-derived fungus *Talaromyces rugulosus*. *Mar. Drugs* **2017**, *15*, 359, DOI: 10.3390/md15110359.

8. Gomi, S.; Imamura, K.-i.; Yaguchi, T.; Kodama, Y.; Minowa, N.; Koyama, M. PF1018, a novel insecticidal compound produced by *Humicola* sp. *J. Antibiot.* **1994**, *47*, 571-580, DOI: 10.7164/antibiotics.47.571.

9. Brady, S.F.; Wagenaar, M.M.; Singh, M.P.; Janso, J.E.; Clardy, J. The cytosporones, new octaketide antibiotics isolated from an endophytic fungus. *Org. Lett.* **2000**, *2*, 4043-4046; DOI: 10.1021/ol006680s.

10. Koizumi, Y.; Arai, M.; Tomoda, H.; Ōmura, S. Oxaline, a fungal alkaloid, arrests the cell cycle in M phase by inhibition of tubulin polymerization. *Biochim. Biophys. Acta* **2004**, *1693*, 47-55, DOI: 10.1016/j.bbamcr.2004.04.013.

11. Shan, W.-G.; Ying, Y.-M.; Yu, H.-N.; Liu, W.-H.; Zhan, Z.-J. Diketopiperazine alkaloids from *Penicillium* spp. HS-3, an endophytic fungus in *Huperzia serrata*. *Helv. Chim. Acta* **2010**, *93*, 772-776, DOI: 10.1002/hlca.200900331.

12. Ogawa, K.; Nakamura, M.; Hayashi, M.; Yaginuma, S.; Yamamoto, S.; Furihata, K.; Shin-Ya, K.; Seto, H. Stachybocins, novel endothelin receptor antagonists, produced by *Stachybotrys* sp. M6222 – II. Structure determination of stachybocins A, B and C. *J. Antibiot.* **1995**, *48*, 1396-1400, DOI: 10.7164/antibiotics.48.1396.

13. Christian, O.E.; Compton, J.; Christian, K.R.; Mooberry, S.L.; Valeriote, F.A.; Crews, P. Using jasplakinolide to turn on pathways that enable the isolation of new chaetoglobosins from *Phomospis asparagi*. *J. Nat. Prod.* **2005**, *68*, 1592-1597, DOI: 10.1021/np050293f.

14. Roll, D.M.; Manning, J.K; Carter, G.T. Hongoquercins A and B, new sesquiterpenoid antibiotics: isolation, structure elucidation, and antibacterial activity. *J. Antibiot.* **1998**, *51*, 635-639, DOI: 10.7164/antibiotics.51.635.

15. Hu, W.; Ohyama, S.; Hasumi, K. Activation of fibrinolysis by SMTP-7 and -8, novel staplabin analogs with a pseudosymmetric structure. *J. Antibiot.* **2000**, *53*, 241-247, DOI: 10.7164/antibiotics.53.241.

16. Isaka, M.; Chinthanom, P.; Kongthong, S.; Supothina, S.; Ittiworapong, P. Hamigeromycins C–G, 14-membered macrolides from the fungus *Hamigera avellanea* BCC 17816. *Tetrahedron* **2010**, *66*, 955-961, DOI: 10.1016/j.tet.2009.11.101.

17. Horak, R.M.; Steyn, P.S.; Vleggaar, R. Metabolites of *Aspergillus ustus*. Part 3. Structure elucidation of austalides G-L. *J. Chem. Soc. Perkin Trans. 1* **1985**, 363-367, DOI: 10.1039/P19850000363.

18. Nishida, H.; Tomoda, H.; Cao, J.; Okuda, S.; Ōmura, S. Purpactins, new inhibitors of acyl-CoA:cholesterol acyltransferase produced by *Penicillium purpurogenum* – II. Structure elucidation of purpactins A, B, and C. *J. Antibiot.* **1991**, *44*, 144-151, DOI: 10.7164/antibiotics.44.144.

19. Kobayashi, A.; Hino, T.; Yata, S.; Itoh, T.J.; Sato, H.; Kawazu, K. Unique spindle poisons, curvularin and its derivatives, isolated from *Penicillium* species. *Agric. Biol. Chem.* **1988**, *52*, 3119-3123, DOI: 10.1271/bbb1961.52.3119.

20. Cueto, M.; MacMillan. J.B.; Jensen, P.R.; Fenical, W. Tropolactones A–D, four meroterpenoids from a marine-derived fungus of the genus *Aspergillus*. *Phytochemistry* **2006**, *67*, 1826-1831, DOI: 10.1016/j.phytochem.2006.01.008.

21. Ling, K.H.; Liou, H.-H.; Yang, C.-M.; Yang, C.-K. Isolation, chemical structure, acute toxicity, and some physicochemical properties of territrem C from *Aspergillus terreus*. *Appl. Environ. Microbiol.* **1984**, *47*, 98-100.

22. Isaka, M.; Yangchum, A.; Intamas, S.; Kocharin, K.; Gareth Jones, E.B.; Kongsaeree, P.; Prabpai, S. Aigialomycins and related polyketide metabolites from the mangrove fungus *Aigialus parvus* BCC 5311. *Tetrahedron* **2009**, *65*, 4396-4403, DOI: 10.1016/j.tet.2009.03.050.

23. Weber, H.A.; Baenziger, N.C.; Gloer, J.B. Podosporin A: a novel antifungal metabolite from the coprophilous fungus *Podospora decipiens* (Wint.) Niessl. *J. Org. Chem.* **1988**, *53*, 4567-4569, DOI: 10.1021/jo00254a028.

24. Bugni, T.S.; Janso, J.E.; Thomas Williamson, R.; Feng, X.; Bernan, V.S.; Greenstein, M.; Carter, G.T.; Maiese, W.M.; Ireland, C.M. Dictyosphaeric acids A and B: new decalactones from an undescribed *Penicillium* sp. obtained from the alga *Dictyosphaeria versluyii*. *J. Nat. Prod.* **2004**, *67*, 1396-1399, DOI: 10.1021/np049973t.

25. Zheng, C.-J.; Kim, C.-J.; Bae, K.S.; Kim, Y.-H.; Kim, W.-G. Bionectins A-C, epidithiodioxopiperazines with anti-MRSA activity, from *Bionectra byssicola* F120. *J. Nat. Prod.* **2006**, *69*, 1816-1819, DOI: 10.1021/np060348t.

26. TePaske, M.R.; Gloer, J.B.; Wicklow, D.T.; Dowd, P.F. Tubingensin A: an antiviral carbazole alkaloid from the sclerotia of *Aspergillus tubingensis*. *J. Org. Chem.* **1989a**, *54*, 4743-4746, DOI: 10.1021/jo00281a010.

27. TePaske, M.R.; Gloer, J.B. The structure of tubingensin B: a cytotoxic carbazole alkaloid from the sclerotia of *Aspergillus tubingensis*. *Tetrahedron Lett.* **1989b**, *30*, 5965-5968, DOI: 10.1016/S0040-4039(01)93829-8.

28. Shiomi, K.; Uchida, R.; Inokoshi, J.; Tanaka, H.; Iwai, Y.; Ōmura, S. Andrastins A~C, new protein farnesyltransferase inhibitors, produced by *Penicillium* sp. FO-3929. *Tetrahedron Lett.* **1996**, *37*, 1265-1268, DOI: 10.1016/0040-4039(95)02412-3.

29. Roy, K.; Chatterjee, S.; Deshmukh, S.K.; Vijayakumar, E.K.S.; Ganguli, B.N.; Fehlhaber, H.-W. Orbuticin, a new secondary metabolite from *Acremonium butyri*. J. Antibiot. 1996, 49, 1186-1187, DOI: 10.7164/antibiotics.49.1186.

30. Jamieson, W.D.; Rahman, R.; Taylor, A. Sporidesmins. Part VIII. Isolation and structure of sporidesmin-D and sporidesmin-F. *J. Chem. Soc. C* **1969**, *11*, 1564-1567, DOI: 10.1039/J39690001564.

31. Perry, D.A.; Maeda, H.; Tone, J. Anti-parasitic compound. *U.K. Patent Application* **1991**, 2 240 100.

32. Ishikawa, Y.; Morimoto, K.; Iseki, S. Atrovenetin as a potent antioxidant compound from *Penicillium* species. *J. Am. Oil Chem. Soc.* **1991**, *68*, 666-668, DOI: 10.1007/BF02662291.

33. Schmitz, F.J.; Bloor, S.J. Xesto- and halenaquinone derivatives from a sponge, *Adocia* sp., from Truk Lagoon[1]. *J. Org. Chem.* **1988**, *53*, 3922-3925, DOI: 10.1021/jo00252a007.

34. M.S.C.; Pedras, C.; Biesenthal, C.J. Isolation, structure determination, and phytotoxicity of unusual dioxopiperazines from the phytopathogenic fungus *Phoma lingam*. *Phytochemistry* **2001**, *58*, 905-909, DOI: 10.1016/S0031-9422(01)00348-X.

35. Oikawa, H.; Murakami, Y.; Ichihara, A. 20-ketoreductase activity of chaetoglobosin A and prochaetoglobosins in a cell-free system of *Chaetomium subaffine* and the isolation of new chaetoglobosins. *Biosci. Biotechnol. Biochem.* **1993**, *57*, 628-631, DOI: 10.1271/bbb.57.628.

36. Kyriakidis, N.; Waight, E.S.; Day, J.B.; Mantle, P.G. Novel metabolites from *Penicillium crustosum*, including penitrem E, a tremorgenic mycotoxin. *Appl. Environ. Microbiol.* **1981**, *42*, 61-62.

37. Liu, W.; Gu, Q.; Zhu, W.; Cui, C.; Fan, G. Two new benzoquinone derivatives and two new bisorbicillinoids were isolated from a marine-derived fungus *Penicillium terrestre*. *J. Antibiot.* **2005**, *58*, 441-446, DOI: 10.1038/ja.2005.57.

38. Lin, Z.-J.; Lu, Z.-Y.; Zhu, T.-J.; Fang, Y.-C.; Gu, Q.-Q.; Zhu, W.-M. Penicillenols from *Penicillium* sp. GQ-7, an endophytic fungus associated with *Aegiceras corniculatum*. *Chem. Pharm. Bull.* **2008**, *56*, 217-221, DOI: 10.1248/cpb.56.217.

39. Kuno, F.; Otoguro, K.; Shiomi, K.; Iwai, Y.; Ōmura, S. Arisugacins A and B, novel and selective acetylcholinesterase inhibitors from *Penicillium* sp. FO-4259 – I. Screening, taxonomy, fermentation, isolation and biological activity. *J. Antibiot.* **1996**, *49*, 742-747, DOI: 10.7164/antibiotics.49.742.

40. Penn, J.; Biddle, J.R.; Mantle, P.G.; Bilton, J.N.; Sheppard, R.N. Pennigritrem, a naturally-occurring penitrem A analogue with novel cyclisation in the diterpenoid moiety. *J. Chem. Soc. Perkin Trans. 1* **1992**, *1*, 23-26, DOI: 10.1039/P19920000023.

41. Kralj Cigić, I.; Prosen, H. An overview of conventional and emerging analytical methods for the determination of mycotoxins. *Int. J. Mol. Sci.* **2009**, *10*, 62-115, DOI: 10.3390/ijms10010062.

42. Suzuki, S.; Hosoe, T.; Nozawa, K.; Kawai, K.-i.; Yaguchi, T.; Udagawa, S.-i. Antifungal substances against pathogenic fungi, talaroconvolutins, from *Talaromyces convolutes*. J. Nat. Prod. 2000, 63, 768-772, DOI: 10.1021/np990371x.
